# Theta synchronization coordinates ventral temporal and medial temporal coding of human face familiarity

**DOI:** 10.1016/j.celrep.2026.118014

**Published:** 2026-09-17

**Authors:** Yilin Li, Jie Zhang, Yue Wang, Hohyun Cho, Peter Brunner, Jasmine Thum, Jon T. Willie, Runnan Cao, Shuo Wang

**Affiliations:** 1 Department of Biomedical Engineering, Washington University in St. Louis, St. Louis, MO 63110, USA; 2 Department of Neurosurgery, Washington University in St. Louis, St. Louis, MO 63110, USA; 3 Department of Radiology, Washington University in St. Louis, St. Louis, MO 63110, USA; 4 Department of Neurosurgery, University of Alabama at Birmingham, Birmingham, AL 35294, USA; 5 Department of Neurosurgery, University of Texas at Austin, Austin, TX 78712, USA; 6 Senior author; 7 These authors contributed equally; 8 Lead contact

## Abstract

Recognizing familiar faces is a fundamental social function that depends on interactions between perceptual and memory systems, yet the underlying neural mechanisms remain poorly understood. Here, we combine single-neuron recordings, intracranial electroencephalography (iEEG), and multi-scale connectivity analyses in the human ventral temporal cortex (VTC) and medial temporal lobe (MTL) to investigate the neural dynamics underlying face familiarity. Theta-frequency phase locking of MTL neurons is associated with face familiarity and familiarization, complementing firing-rate signals. The VTC represents visual features through an axis-based code that disproportionately contributes to familiarity representations. Familiarity is further associated with enhanced theta synchronization between the VTC and MTL, particularly between VTC visual-feature-coding and MTL familiarity-coding channels. Enhanced theta-frequency spike-iEEG coherence for familiar faces suggests that MTL phase locking is coordinated with large-scale VTC activity. Together, these findings identify theta synchronization as a key mechanism coordinating the neural dynamics underlying face familiarity processing in humans.

## In brief

Li et al. reveal how the human brain recognizes familiar faces by coordinating visual perception and memory through theta-frequency synchronization. Their findings show that rhythmic neural activity links feature representations in the visual cortex with memory signals in the medial temporal lobe, providing a mechanistic framework for processing face familiarity.

## Graphical abstract

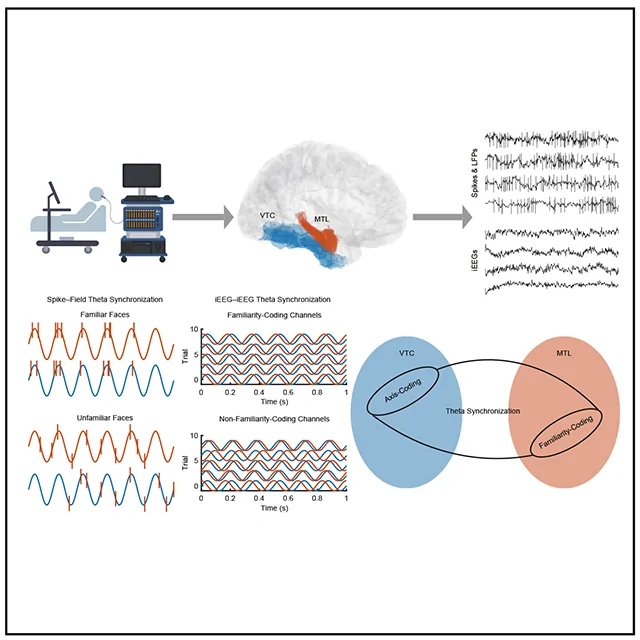

## INTRODUCTION

Recognizing familiar faces is a cornerstone of human social interaction, making familiarity a central topic in the field of face perception since the 1970s.^1,2^ Familiarity—getting to know someone—is typically acquired through repeated visual exposure to the same identity across multiple occasions, despite tremendous variability in viewpoint, expression, hairstyle, makeup, and age.^3^ Impairments in recognizing familiar faces have been reported in various neurological conditions, including prosopagnosia, amnesia, and Capgras delusion, often linked to lesions in the ventral temporal cortex (VTC) and medial temporal lobe (MTL).^4–6^ These findings suggest that both perceptual and memory-driven processes contribute to familiar face recognition. However, the precise neural underpinnings of familiarity remain unclear, despite extensive neuroimaging studies conducted over the past two decades.^7^ A key open question is how the neural representations of familiar faces differ from those of unfamiliar faces along the ventral visual pathway. In other words, how are familiarity-related signals coordinated between the visual cortex and higher-order mnemonic regions to support generalized recognition of previously encountered faces?

Behavioral research has consistently demonstrated that familiar faces are perceived more efficiently than unfamiliar faces, with faster and more robust identification across changes in viewpoint, expression, and context, leading to arguments for a qualitatively distinct processing system.^8–11^ In contrast, neuroimaging studies have yielded mixed findings regarding the neural substrates of familiarity, depending on how familiarity is defined, the experimental paradigm, recording approach, and analytic methods.^7,10,12^ Specifically, regions within the extended faceprocessing system,^13,14^ including the anterior temporal lobe (ATL) and MTL, are consistently reported to encode knowledge-based familiarity information.^15–17^ Our recent study has also shown that single neurons in the human MTL encode face familiarity.^18^ Discrepancies primarily arise in the core face-processing areas, such as the occipital face area (OFA) and fusiform face area (FFA) within the VTC, which are often reported as irrelevant in studies of purely visual familiarity^19–21^ but show significant involvement when familiarity is probed using famous or personally familiar faces.^11,22–24^ Meanwhile, intracranial electroencephalography (iEEG) studies suggest that neural processing of familiar faces begins to diverge from that of unfamiliar faces at approximately 250 ms after stimulus onset.^7^ Collectively, these findings indicate that the neural correlates of familiarity are distributed across the face-processing system and likely unfold through specific spatiotemporal dynamics.

While both the VTC and MTL contribute to familiar face recognition, it remains unclear how neural dynamics are coordinated between these regions during face familiarity processing. Face identity information can be decoded from neural responses in both the VTC and MTL for familiar and unfamiliar faces, albeit with varying performance.^18,25–27^ Recent intracranial neurophysiology studies suggest distinct coding schemes in the VTC and MTL that explain how the brain extracts high-level identity information from visual images.^28,29^ Under this hypothesis, the VTC encodes faces via an axis-based coding model, whereby a given face is represented by a population of neurons, each tuned to one or a few specific visual feature axes (e.g., texture, shape).^29,30^ In contrast, the MTL encodes identities using both an exemplar-based code^31,32^ and a nonlinear, receptive-field-like code.^28,33^ Interestingly, familiar faces are represented more distinctly than unfamiliar faces in both the axis-coding VTC^24,34^ and the exemplar-coding MTL.^18,35^ Together, these results suggest a transition in face coding from distributed, feature-based representations in the higher visual cortex to knowledge-based representations in the MTL. Yet it remains unclear how familiarity-related neural activity is coordinated along the ventral visual pathway and whether or how familiarity influences neural representations in these regions to enhance identity distinctiveness.

In the present study, we recorded single-neuron activity and local field potentials (LFPs) from the MTL, together with iEEG from both the VTC and MTL, while participants viewed 500 naturalistic face images from 50 celebrities. For each identity, 10 different images were included to probe image-invariant face processing. Following the recordings, participants completed a questionnaire indicating whether they were familiar with each identity. To characterize the neural dynamics underlying face familiarity across spatial scales, we performed a series of complementary synchronization analyses spanning local neuronal activity, regional population activity, and long-range interareal interactions. First, at the microscopic scale, we examined whether pairwise phase consistency (PPC) between single-neuron spiking and local theta oscillations in the MTL encoded face familiarity and short-term familiarization induced by repeated visual exposure. Second, at the regional population level, we characterized familiarity representations across VTC and MTL subregions using iEEG recordings. Finally, at the network level, we investigated how face familiarity modulated functional interactions between the VTC and MTL using multiple complementary measures, including phase-locking value (PLV), Granger causality (GC), and spike-iEEG synchronization, thereby linking local neuronal dynamics with large-scale cortical coordination. Together, these analyses reveal that theta synchronization provides a common organizing principle across multiple spatial scales, coordinating familiarity-related neural dynamics from local spike-field coupling within the MTL to long-range interactions between perceptual representations in the VTC and familiarity-related representations in the MTL.

## RESULTS

### Behavior

Twenty-two neurosurgical patients (51 sessions; Table S1) viewed 500 unique natural face images of 50 celebrities from the CelebA dataset (10 images per celebrity) while performing a one-back task (Figure 1A; accuracy = 77.57% ± 21.28% [mean ± SD across sessions]). After completing the recordings, patients completed a questionnaire indicating whether they recognized each face identity (one of the 10 faces was randomly selected from each identity for this questionnaire), independent of name retrieval. We refer to the face identities that a patient recognized as *familiar* and the face identities that a patient did not recognize as *unfamiliar*. On average, patients rated 42.1% ± 19.4% (mean ± SD across patients) of the identities shown as familiar.

### Theta-frequency phase locking of single neurons in the human MTL encodes face familiarity

We recorded single-neuron activity and LFPs using microwires implanted in the amygdala (868 neurons) and hippocampus (1,044 neurons) of 16 patients (40 sessions; Table S1). Consistent with our previous findings,^18^ a significant proportion of neurons in both the amygdala (*n* = 56, 6.45%; binomial test against 5% chance level: *p* = 0.02) and hippocampus (*n* = 75, 7.18%; binomial *p* = 8.72 × 10^−4^) significantly differentiated familiar versus unfamiliar faces (two-tailed two-sample *t* test, *p* < 0.05; Figure S1). Furthermore, we found that a substantial proportion of amygdala neurons (559 neurons, 64.4%; binomial *p* < 10^−20^; see Figure 1B for an example) and hippocampal neurons (653 neurons, 62.6%; binomial *p* < 10^−20^; see Figure 1C for an example) exhibited significant phase locking to local theta oscillations (see Figure S2A for the distribution of phase angles).

We next assessed how much the spikes of single neurons were phase locked to the ongoing theta oscillations by calculating PPC across all spike-LFP pairs (see STAR Methods). Notably, we found that the PPC of spike-LFP pairs in the theta frequency range was significantly greater for familiar faces than for unfamiliar faces in both the amygdala (Figures 1D and 1E; two-tailed paired *t* test: *t*(3711) = 2.70, *p* = 0.007, *d* = 0.04, 95% confidence interval [CI] = [1.70 × 10^−4^, 0.001]) and hippocampus (Figures 1D and 1E; *t*(3593) = 3.18, *p* = 0.002, *d* = 0.05, 95% CI = [2.41 × 10^−4^, 0.001]). However, there were no significant differences in phase angles between familiar and unfamiliar faces (permutation test: *p* > 0.05 for both the amygdala and hippocampus; Figure S2A). We obtained similar findings using all spike-LFP pairs without selection (as shown in Figure 1) and spike-LFP pairs with significant phase locking, and we further confirmed that different PPC methods and parameters yielded robust and consistent results. Therefore, in addition to the firing-rate-based mechanism differentiating familiar and unfamiliar faces, as reported in our previous study,^18^ we further identified differences in theta synchronization between familiar and unfamiliar faces.

In addition, spectral parameterization using the FOOOF toolbox^36^ confirmed the presence of oscillation-specific activity in the low-frequency (delta–theta) range after separating periodic and aperiodic spectral components (Figures S2B and S2C). Importantly, the observed familiarity-related PPC effects could not be explained by differences in theta-band power (Figure S2B) or in the oscillation-specific (periodic) component of the theta spectrum (Figure S2C). Instead, they likely reflect changes in spike-phase alignment relative to ongoing theta oscillations.

Together, these results demonstrate that theta-frequency phase locking of single neurons in the human MTL is associated with face familiarity, complementing rate-based signals and highlighting theta synchronization as a key mechanism underlying face familiarity processing.

### Theta-frequency phase locking of single neurons in the human MTL encodes face familiarization

Familiarity with a human face typically develops through repeated visual encounters during social interactions. Next, we investigated whether theta synchronization exhibits exposure-dependent changes similar to those observed in firing-rate-based representational distance measures,^18^ which have been linked to pattern-separation-like effects. Indeed, a significant proportion of MTL neurons (162/910, 17.8%, binomial *p* < 10^−20^) and spike-LFP pairs (238/3508, 6.78%, binomial *p* = 1.61 × 10^−6^) with significant theta phase locking showed a significant change in PPC as a function of identity exposure. Interestingly, similar to the firingrate-based representational distance measure, we observed both spike-LFP pairs with PPC that linearly increased with identity exposure (104, 43.7%; Figures 2A and 2B) and those with PPC that linearly decreased with identity exposure (134, 56.3%; Figures 2C and 2D). Furthermore, as with firing rate,^18^ we observed similar results for both familiar and unfamiliar faces. In addition, the PPC changes associated with repeated exposure could not be explained by changes in theta power (Figure S2D) or oscillation-specific theta activity (Figure S2E), indicating that familiarization primarily modulates spike-phase alignment rather than oscillatory amplitude.

Importantly, the linear change in PPC as a function of identity exposure was independent of changes in firing rate. Specifically, the correlation between firing rate and identity exposure was not significant in neurons exhibiting a significant PPC correlation (neurons with a positive PPC correlation: *r*(500) = −0.03 ± 0.45 [mean ± SD across neurons]; two-tailed one-sample *t* test against 0: *t*(79) = −0.52, *p* = 0.60, *d* = −0.06, 95% CI = [−0.13, 0.07]; neurons with a negative PPC correlation: *r*(500) = −0.05 ± 0.52, *t*(83) = −0.83, *p* = 0.41, *d* = −0.09, 95% CI = [−0.16, 0.07]), suggesting that theta synchronization represents a distinct neural mechanism for face familiarization. Furthermore, neurons showing an exposure-dependent modulation through firing rate (FR neurons) were not more likely to also show such modulation through PPC (PPC neurons). In other words, the proportion of PPC neurons within FR neurons (*n*_Both_/*n*_FR_) was not significantly higher than the proportion of PPC neurons within all neurons (*n*_PPC_/*n*_All_; χ^2^ test: *p* = 0.70; Figure 2E). This indicates that the overlap between the two groups of neurons was not higher than expected by chance, further supporting the conclusion that they represent independent neural populations encoding face familiarization. Lastly, the exposure-dependent neural modulation observed through firing rate was not correlated with that observed through PPC (*r*(910) = −0.04, *p* = 0.22; Figure 2F), and the two groups of neurons appeared to constitute largely distinct populations (Figure 2F).

We obtained qualitatively similar results when the amygdala (neurons: 92/471, 19.53%, binomial *p* < 10^−^; spike-LFP pairs: 128/2043, 6.27%, binomial *p* = 0.005; Figures S2F and S2H) and hippocampus (neurons: 70/439, 15.95%, binomial *p* < 10^−20^; spike-LFP pairs: 110/1465, 7.51%, binomial *p* = 1.43 × 10^−5^; Figures S2G and S2I) were analyzed separately. Specifically, we observed that PPC linearly increased with identity exposure in 52 amygdala (40.63%; Figure S2F) and 52 hippocampal (47.27%; Figure S2G) spike-LFP pairs and linearly decreased with identity exposure in 76 amygdala (59.38%; Figure S2H) and 58 hippocampal (52.72%; Figure S2I) spike-LFP pairs. Thus, both increasing and decreasing PPC effects were present in each structure with comparable proportions. Furthermore, some participants performed the task in more than one session (Table S1), and we confirmed that the familiarization effect was observed in both the first and subsequent sessions (Figures S2J–S2M). Finally, we fit linear regression models to predict the familiarization effect from firing rate alone or from both firing rate and PPC within neurons exhibiting significant PPC modulation. We found that the model including PPC explained significantly more variance than the model based on firing rate alone (Δ*R*^2^ = 0.17, permutation *p* < 0.001).

Together, these results demonstrate that theta-frequency synchronization in the human MTL is associated with face familiarization independently of firing-rate-based mechanisms, revealing a complementary neural mechanism that supports the gradual familiarization of faces.

### Familiarity coding in the VTC and MTL

We simultaneously recorded iEEG activity from the MTL (amygdala: 137 channels; hippocampus: 113 channels; see Figure 3A for channel distribution) and the VTC (fusiform gyrus [FG]: 182 channels; inferior temporal gyrus [ITG]: 231 channels) in 13 patients to investigate how face familiarity is encoded in these brain regions (Table S1). We extracted high-gamma power (HGP; 70–170 Hz) from the iEEG signals for further analysis^29,38–40^ (see Figures S3A–S3C for analyses across different frequency ranges). We found that 26 channels (6.3%) in the VTC (11 in the FG and 15 in the ITG; see Figure 3B for representative channels and Figure S3D for group results) and 11 channels (4.4%) in the MTL (8 in the amygdala and 3 in the hippocampus; Figure 3B and Figure S3D) exhibited significantly different responses to familiar versus unfamiliar faces. In the majority (75.68%) of these channels, familiar faces elicited stronger responses than unfamiliar faces (Figure S3D). Moreover, we obtained qualitatively similar results when using an equal number of familiar and unfamiliar faces within each participant (Figures S4A and S4B). We also obtained qualitatively similar results when the gender (Figure S4C) and race (Figure S4D) compositions of the familiar and unfamiliar faces were matched. Interestingly, the neural representational distance for familiarity—defined as the absolute difference in population distance between familiar and unfamiliar faces (see STAR Methods for details)—increased from the VTC to the MTL (Figure 3C; two-tailed paired *t* test: *t*(19) = −2.20, *p* = 0.04, *d* = −0.492, 95% CI = [−0.229, −0.006]), suggesting that familiar identities are more neurally distinct in the MTL than in the VTC. These findings indicate that familiarity-mediated pattern separation becomes more pronounced in mnemonic MTL areas than in the higher-order visual cortex.

Furthermore, we calculated the coupling between theta-band power and HGP in familiarity-coding channels. Trial-by-trial correlations between theta-band power and HGP were generally weak to moderate and predominantly negative, indicating that increased theta power tended to co-occur with reduced high-gamma activity (Wilcoxon signed-rank test: *p* = 0.0056; Figure S4E). This pattern was observed for both familiar (Figure S4F) and unfamiliar (Figure S4G) faces, but interestingly, the correlations were significantly more negative for unfamiliar than for familiar faces (Wilcoxon signed-rank test: *p* = 0.013).

Our recent study demonstrated that the human VTC encodes faces using an axis-based feature code.^29^ To examine whether familiarity interacts with this feature coding, we identified axiscoding channels in the VTC. Specifically, we extracted visual features from each face image using the pre-trained deep neural network VGG-16 and modeled the neural responses of each VTC channel to these features with partial least squares (PLS) regression (see STAR Methods for details). Consistent with our previous findings,^29^ we observed a significant proportion of axis-coding channels in the FG (28.13%, *n* = 36, binomial test: *p* < 10^−16^; see Figure 3D for the distribution of axis-coding channels and Figure 3F for example channels), but not in the ITG (5.4%, *n* = 4, *p* = 0.51), among visually responsive channels. While axis-coding channels contained significantly more familiarity-coding channels compared to the whole population (*n*_Both_/*n*_Axis-Coding_ = 15.0%; *n*_Familiarity-Coding_/*n*_All_ = 6.54%; χ^2^ test: 6.42 × 10^−4^; Figure 3E), familiar faces showed comparable alignment with the most-preferred feature axis relative to unfamiliar faces (see Figure 3F for example channels; Pearson correlation: familiar faces: *r*(40) = 0.40 ± 0.08 [mean ± SD across channels]; unfamiliar faces: *r*(40) = 0.38 ± 0.05; two-tailed paired *t* test: *t*(39) = 1.20, *p* = 0.24, *d* = 0.19, 95% CI = [−0.012, 0.048]), indicating that face familiarity did not modulate axis coding in the VTC.

Together, these findings show that the VTC primarily encodes visual features through an axis-based code, while axis-coding channels disproportionately contribute to familiarity coding.

### Familiarity enhances synchronization between the VTC and MTL at the mesoscopic scale

How do the VTC and MTL interact during familiarity encoding? Prior studies have demonstrated robust effective and anatomical connectivity between the VTC and MTL.^41–43^ Here, we examined functional connectivity both along the VTC-MTL pathway and within the MTL locally.

We first computed the phase-locking value (PLV) between all possible channel pairs, each consisting of one VTC channel in the FG or ITG and one channel in the amygdala or hippocampus (see STAR Methods for details). Across all subregion combinations, VTC-MTL pairs showed significant synchronization relative to null distributions at low frequencies (<10 Hz; see Figure 4A for PLV across frequencies), for both familiarity-coding and non-familiarity-coding channels in the MTL.

We then compared the PLV between VTC channels paired with MTL familiarity-coding channels and those paired with MTL non-familiarity-coding channels. Familiarity-coding channels in the MTL exhibited stronger theta synchronization with both FG (*p* = 0.053; see STAR Methods for statistical procedures) and ITG (*p* = 0.063) channels within the theta frequency range (3–8 Hz; Figure 4A) compared to non-familiarity-coding channels in the MTL. Familiarity-coding channels in both the FG and MTL exhibited significantly stronger theta synchronization than non-familiarity-coding channels in these areas (*p* = 0.026). Furthermore, when breaking down for subregions (Figure 4A), we found that familiarity-coding channels in the amygdala exhibited significantly stronger theta synchronization with FG channels within the theta frequency range (*p* = 0.031; FG-hippocampus: *p* = 0.69; ITG-amygdala: *p* = 0.096; ITG-hippocampus: *p* = 0.99).

To probe the temporal dynamics of VTC-MTL synchronization during familiarity processing, we conducted a time-resolved PLV analysis (see STAR Methods for details; Figure 4B). Our results showed that FG channels synchronized with amygdala channels rapidly after stimulus onset, consistent with previous findings.^44^ In line with the above results, familiarity-coding channels in the amygdala exhibited significantly stronger synchronization with FG channels (Figure 4B), particularly in the theta frequency range (Figure 4C; *p* = 0.029; FG-hippocampus: *p* = 0.54; ITG-amygdala: *p* = 0.81; see also Figures 5A–5C), indicating a specific role of the FG-amygdala pathway in processing face familiarity. Interestingly, there was significant desynchronization between the ITG and hippocampus for familiarity-coding channels (*p* = 0.032), indicating that different VTC and MTL subregions may play distinct roles in these processes.

In particular, compared to non-axis-coding channels, axis-coding channels in the VTC exhibited stronger theta synchronization with MTL familiarity-coding channels (Figure 4D; see also Figures 5D–5F). Likewise, compared to non-familiarity-coding channels, familiarity-coding channels in the MTL showed stronger theta synchronization with VTC axis-coding channels (Figure 4E). Therefore, these results point to an interaction between visual feature coding in the VTC and familiarity processing in the MTL, suggesting that axis-based feature coding in the VTC contributes to the neural representation of face familiarity.

To further investigate the direction of VTC-MTL interactions during familiarity coding, we conducted a GC analysis on the same set of channel pairs used in the PLV analysis. Consistent with the PLV results, we observed significant GC between VTC and MTL channels at low frequencies (Figures 4F–4I, left). Notably, familiarity specifically enhanced bidirectional GC between the FG and amygdala (Figure 4F, right; feedforward: *p* = 0.048; feedback: *p* = 0.038), but not between the other channel combinations (Figures 4G–4I, right, all *p* values > 0.05), again indicating that the FG-amygdala pathway may play a unique role in mediating familiarity-related interactions between the VTC and MTL.

We further repeated the analysis in 279/663 channels that had fewer than 5% rejected trials and thus showed less interictal activity, and obtained qualitatively similar results (Figure S5; see discussion).

Together, these findings demonstrate that face familiarity is associated with enhanced theta-band synchronization between the VTC and MTL, with a particularly prominent contribution from the FG-amygdala pathway. The association between axis-based visual feature coding in the VTC and familiarity coding in the MTL is consistent with a contribution of VTC visual representations to familiarity-related neural responses in the MTL. Furthermore, the observed bidirectional functional interactions during face familiarity processing indicate coordinated activity between perceptual and memory-related systems.

### Familiarity modulates spike-iEEG synchronization

At the microscopic scale in the MTL, we observed encoding of face familiarity through both firing rates (Figure S1) and theta synchronization (Figure 1). How do these effects arise from VTC inputs? To address this question, we computed spike-iEEG coherence within the MTL and between the MTL and VTC using PPC, to examine how familiarity-coding neurons in the MTL coordinate with both local and distant neural populations. We then compared PPC values for familiar versus unfamiliar faces to assess how familiarity modulates spike-iEEG coherence.

PPC was significantly greater for familiar than for unfamiliar faces, both locally within the MTL (see Figure 6A for an example where spikes were primarily phase locked to the peak [0°] of theta oscillations for familiar faces but distributed more randomly or uniformly for unfamiliar faces; see Figure 6B for an example where spikes were primarily phase locked to the trough of theta oscillations for familiar faces but evenly distributed for unfamiliar faces; see Figures 6C–6E for group results) and between the MTL and VTC (Figures 6F–6H). Consistent with our earlier findings (Figures 1 and 4), this familiarity-driven enhancement was most pronounced in the theta frequency range (Figures 6C and 6F; two-tailed paired *t* test: MTL spike-MTL iEEG: *t*(1535) = 2.79, *p* = 0.005, *d* = 0.07, 95% CI = [5.38 × 10^−4^, 0.003]; MTL spike-VTC iEEG: *t*(2955) = 2.88, *p* = 0.004, *d* = 0.05, 95% CI = [4.26 × 10^−4^, 0.002]). Time-frequency analysis (Figures 6D and 6G) and PPC time courses (Figures 6E and 6H) within the theta frequency range further illustrated the temporal dynamics of spike-iEEG coherence, showing that familiar faces induced stronger theta-band synchronization both within the MTL and between the MTL and VTC. Consistent results were observed across individual subregions of the MTL and VTC (Figure S6).

Together, these findings reveal enhanced spike-iEEG synchronization during face familiarity processing, consistent with results observed at both microscopic (Figure 1) and mesoscopic (Figure 4) scales. These results highlight the role of functional connectivity across multiple spatiotemporal scales in mediating familiarity processing along the VTC-MTL pathway and suggest that the theta-frequency phase-locking mechanism for face familiarity in single neurons of the human MTL (Figure 1) may, in part, arise from VTC inputs.

## DISCUSSION

In this study, we systematically characterized the neural mechanisms underlying human face familiarity across multiple spatiotemporal scales. We found that theta-frequency phase locking of single neurons in the MTL was associated with both face familiarity and familiarization, complementing firing-rate signals. The VTC primarily represented visual features through an axis-based code, with axis-coding channels disproportionately contributing to familiarity representations. Face familiarity enhanced thetaband synchronization between the VTC and MTL, particularly along the FG-amygdala pathway, supporting bidirectional interactions between perceptual and memory systems. Enhanced spike-iEEG coherence further suggested that theta synchronization of MTL neurons is, in part, driven by inputs from the VTC. Together, these findings identify theta synchronization as a multi-scale mechanism that coordinates neural dynamics between the VTC and MTL during face familiarity processing.

We observed that the modulation of synchronization occurred predominantly at the theta frequency, consistent with the well-established association between theta oscillations and memory.^45,46^ Theta oscillations bring together activity of sensory- and/or memory-activated neurons in time, thereby affecting behavioral output and plasticity.^47^ Our results align with the hypothesis that information integration is achieved through oscillatory synchronization^48^ or communication through coherence (CTC).^49^ On the other hand, our previous study showed that face familiarity and familiarization were associated with increased representational distances among neuronal population responses, driven by alterations in neuronal firing rates rather than oscillatory synchrony.^18^ This aligns with a firing-rate-based mechanism for feature binding into coherent visual representations,^50^ supporting the hypothesis of communication through resonance (CTR).^51^ Importantly, our present findings revealed that the neuronal populations demonstrating spike-LFP synchrony were statistically independent of those showing changes in representational distance.^18^ Therefore, spike-LFP synchrony may serve as an additional mechanism for face familiarity and familiarization. The disentanglement of synchronization neurons and distance-coding neurons suggests that distinct neural populations—and potentially different neuron types—communicate through resonance or coherence, working together to support the process of face familiarization.

In this study, we observed only weak familiarity coding in the VTC. By contrast, the human VTC plays a critical role in encoding visual facial features,^29^ and indeed we found that VTC channels primarily encoded visual features rather than familiarity (Figures 3D and 3E). This finding suggests that the FG mainly supports visual feature processing, whereas familiarity per se may be represented in distributed networks beyond the core perceptual face system, including memory- and association-related regions. This dissociation underscores the importance of distinguishing perceptual encoding from contextual familiarity in models of face recognition. Interestingly, face familiarity did not modulate visual feature coding (Figure 3F), although channels encoding visual features were more likely to also encode familiarity (Figure 3E). Importantly, VTC axis-coding channels were more strongly connected with MTL familiarity-coding channels than were VTC non-axis-coding channels (Figure 4D), and VTC axis-coding channels were also more strongly connected with MTL familiarity-coding channels than with MTL non-familiarity-coding channels (Figure 4E). Together, our study delineates how visual feature coding and familiarity coding are related in the VTC and MTL.

The human amygdala and hippocampus are critical for both face processing and face memory.^52^ Studies using visually familiar faces without biographical or emotional content have demonstrated that familiar faces elicit a distinct amygdala blood-oxygen-level-dependent (BOLD) response relative to that elicited by novel faces.^21^ Unlike the ventral occipitotemporal face-preferential regions, where BOLD activity primarily reflects visual information regardless of face familiarity, the amygdala and hippocampus show a marked increase in BOLD signal once a face is identified as familiar.^15^ Moreover, identity-selective neurons in the human amygdala and hippocampus are more likely to respond to familiar faces than unfamiliar ones.^35^ However, these prior studies primarily focused on neural activation. In contrast, our study reveals familiarity-related neural dynamics characterized by theta-band synchronization.

The Haxby framework for face processing^13^ has motivated numerous neuroimaging studies examining connectivity among face-selective nodes,^41–44,53–55^ yet relatively few have addressed how face familiarity modulates these connections.^41,43,56^ Earlier studies using dynamic causal modeling (DCM) to test effective connectivity proposed a strictly hierarchical serial model,^41^ whereas later work suggested a parallel, reciprocal architecture within and across the core and extended face-processing systems.^42,43^ In particular, the FG is considered a hub of the identity-processing pathway, exerting dominant influence on the extended system, including the ATL, orbitofrontal cortex (OFC), and MTL. However, findings regarding the effects of familiarity remain limited and mixed. For example, Fairhall et al. argued that familiarity specifically modulates FG-OFC connectivity,^41^ whereas a more recent study reported that familiarity enhances connectivity between core face areas and broader semantic networks, including the ATL, MTL, and medial prefrontal cortex (mPFC).^56^ In line with these findings, we consistently observed that familiarity enhanced FG-amygdala synchronization within the theta band across different scales and metrics but not in other VTC-MTL subregion combinations. These results suggest that familiarity strengthens a core limbic-visual link central to identity processing, while engaging broader connectivity in a flexible, task- and region-specific manner.

Findings from neuroimaging studies have highlighted a feedforward-dominant connection within the Haxby framework.^13^ However, such findings rely heavily on DCM, which warrants caution given its dependence on directional assumptions and a restricted model space.^57,58^ Moreover, fMRI-based DCM depends on slower hemodynamic models that cannot capture fast oscillatory synchrony and are prone to signal dropout in the inferior and medial temporal areas critical for face processing.^59,60^ These limitations underscore the need for high-spatiotemporal-resolution neural recordings, such as iEEG, to capture interareal connectivity at a finer temporal scale.^39,61^ To date, only a handful of studies have examined functional connectivity during face processing using direct intracranial recordings.^29,44,62,63^ While these studies have provided insights into time-resolved inter-regional synchronization between face-processing areas, such as the FG and MTL, they have not tested connectivity specifically in the context of familiarity. Nevertheless, recent research has demonstrated the utility of intracranial recordings for reliably assessing interareal interactions in visual memory and learning,^64^ processes directly relevant to face familiarization.

### Limitations of the study

In this study, we define familiarity as contextual familiarity—a construct that requires personal knowledge extending beyond mere visual exposure. This distinction is important, as it moves beyond low-level perceptual familiarity to a more ecologically valid conception of real-world social recognition. By adopting a paradigm that accounts for image independence, we showed that neural representations of familiar faces are not tied to specific visual instances but instead reflect higher-order, context-rich associations. On the other hand, we showed that theta phase locking dynamically encodes face familiarization as a function of visual exposure, in a manner similar to face familiarity. This finding helps address a key limitation of the dichotomous contrast between familiar and unfamiliar faces, as familiarity in everyday life exists on a continuum. Individuals differ in how well they know others, ranging from fleeting encounters to enduring, lifelong partnerships. Future research should therefore aim to capture this graded spectrum of familiarity, enabling a more nuanced understanding of how it is encoded across different levels of personal relevance.

## RESOURCE AVAILABILITY

### Lead contact

Further information and requests for resources should be directed to and will be fulfilled by the lead contact, Shuo Wang (shuowang@wustl.edu).

### Materials availability

This study did not generate new unique reagents.

## STAR★METHODS

### EXPERIMENTAL MODEL AND STUDY PARTICIPANT DETAILS

The detailed methods were described in our previous studies.^18,28,29,65,66^ Below, we summarize the essential methods relevant to the present study and elaborate the methods unique to it.

There were 51 sessions from 22 patients in total (Table S1). We only included patients who indicated whether they recognized each face identity in a follow-up questionnaire.^18,28^ All participants provided written informed consent, and the study protocol was approved by the Institutional Review Boards of Washington University in St. Louis (WUSTL; protocol number: 202201019) and West Virginia University (WVU; protocol number: 1709745061).

Parts of the dataset analyzed in this study overlap with previously published recordings from our laboratory,^18,28,29,65^ while additional recordings were newly collected for the present work. Specifically, the dataset includes recordings from 14 previously reported patients and 8 newly recruited patients. Previous studies using subsets of these data focused primarily on firing-rate-based familiarity coding by single neurons in the human MTL,^18^ identity coding and region-based feature coding in the human MTL,^28^ the computational pathway linking axis coding in the VTC and region coding in the MTL,^29^ and the public release of a single-neuron dataset for face perception.^65^ In contrast, the present study addresses a distinct question by investigating familiarity-related neural synchronization and network interactions across the VTC and MTL. The synchronization, connectivity, and multi-scale network analyses of familiarity processing presented here have not been reported previously.

### METHOD DETAILS

#### Stimuli

We used faces of celebrities from the CelebA dataset.^67^ We selected 50 identities (individuals). For each, we picked 10 different images, resulting in a total of 500 images. The selected stimuli included both genders (33 of the 50 identities were male) and multiple races (40 identities were Caucasian, 9 identities were African American, and 1 identity was biracial). The same stimuli were used for all sessions. It is worth noting that we used different pictures of the same identity to investigate face familiarization (Figure 1A). This not only provided better generalizability but also mimicked real world conditions in which people do not repeatedly view an identical face but people perceive the same face identity with different appearances. Using different pictures of the same identity may also reduce stimulus suppression and adaptation.

We confirmed that familiar and unfamiliar faces did not significantly differ in low-level visual features, including luminance (i.e., luminance after converting RGB values to the CIE 1976 L*, u*, v* color space; two-tailed paired *t* test across participants: *p* > 0.05) and wavelength (mean hue value after converting RGB to hue, saturation, and value [HSV]; see OSF: https://osf.io/auzjy/ for detailed definition and implementations; *p* > 0.05). There were 31.69% ± 7.88% (mean ± SD across participants) familiar female faces and 35.27% ± 5.72% unfamiliar female faces (*p* = 0.32), 72.69% ± 8.34% familiar Caucasian faces and 84.09% ± 3.56% unfamiliar Caucasian faces (*p* = 0.0012), and 24.57% ± 8.68% familiar African American faces and 14.66% ± 3.4% unfamiliar African American faces (*p* = 0.0037). Although there was a significant difference in race between familiar and unfamiliar faces, we obtained qualitatively similar results when the race composition of the familiar and unfamiliar faces was matched (Figures S4C and S4D; and likewise for gender, even though no significant difference in gender composition was observed).

Face familiarity is not a binary construct but is inherently graded, spanning levels from perceptual recognition to name retrieval and richer semantic knowledge. Accordingly, it is possible that some faces categorized as unfamiliar in the present study were in fact weakly familiar at a semantic level, which could lead to an underestimation of the true proportion of familiar faces. Importantly, such imprecision would be expected to reduce sensitivity rather than generate spurious familiarity effects. Consistent with this interpretation, prior work using the same recognition-based procedure has revealed robust neural signatures of face familiarity.^18^ Future studies that explicitly dissociate graded levels of familiarity and semantic knowledge will be critical for elucidating how different degrees of person knowledge modulate neural coding across perceptual and memory systems.

An important consideration is that face familiarity is a multidimensional construct that extends beyond perceptual recognition. In the present study, familiarity was operationalized through participants’ recognition of celebrity identities, a commonly used approach in the face-processing literature. However, familiar and unfamiliar faces likely differ along multiple dimensions that covary with familiarity, including semantic knowledge, emotional significance, autobiographical associations, attentional relevance, salience, arousal, and predictability. Although we controlled a range of low-level visual properties and examined familiarity-related effects across multiple neural measures, our design does not isolate familiarity as a single computational variable or dissociate it from these naturally co-occurring factors. Consequently, the observed neural responses should be interpreted as reflecting the broader representational state associated with real-world face familiarity rather than a pure familiarity signal independent of other cognitive and affective processes. Importantly, the one-back task imposed identical behavioral demands for familiar and unfamiliar faces, minimizing explicit differences in task engagement or decision-making. Nevertheless, we cannot exclude the possibility that familiar faces automatically recruit greater attention or emotional significance because of their behavioral relevance. Such effects are intrinsic to natural face familiarity rather than artifacts of the experimental task and likely contribute to the neural dynamics observed here. Future studies combining experimentally learned identities, longitudinal familiarization paradigms, explicit measurements of semantic and emotional associations, and orthogonal manipulations of attention and salience will be important for dissociating these interacting components and determining their respective contributions to familiarity-related neural synchronization.

A related limitation concerns our use of the term ‘‘familiarization’’. While repeated exposure to initially unfamiliar identities produced measurable changes in neural representations and representational geometry, the manipulation was conducted within a single experimental session and therefore differs substantially from the prolonged and ecologically rich learning processes through which face familiarity is typically acquired in everyday life. Therefore, the observed effects do not reflect the full development of real-world familiarity. Instead, our findings should be interpreted as evidence that short-term exposure can induce measurable familiarity-related neural changes, providing a tractable experimental model for studying the early stages of familiarity formation. Whether the same neural mechanisms scale to the acquisition of long-term, socially meaningful familiarity remains an important question for future research.

#### Experimental procedure

We used a one-back task. On each trial, a single face was presented at the center of the screen for 1 s, followed by a uniformly jittered inter-stimulus interval (ISI) of 0.5–0.75 s (Figure 1A). Each image subtended a visual angle of approximately 10°. Patients pressed a button if the current face image was identical to the immediately preceding image. One-back repetitions comprised 9–10% of trials. Each face was shown once unless repeated in one-back trials, and responses from one-back trials were excluded to ensure an equal number of responses for each face. This task maintained attention to the faces while avoiding potential biases associated with focusing on specific facial features (e.g., as might occur if patients were asked to judge a particular feature). The order of face presentation was randomized for each patient.

Behavioral accuracy was computed from infrequent one-back catch trials that served solely as an attentional monitoring procedure. Because these trials constituted only approximately 10% of all trials, occasional missed button presses likely reflect lapses in motor responding rather than failures to attend to the stimuli. Consistent with this interpretation, simultaneous eye-tracking measurements confirmed sustained fixation and visual engagement throughout the task.^68^ Importantly, all one-back trials were excluded from the neural analyses reported in this study.

Although the behavioral task consisted of a simple one-back attention-monitoring paradigm, our intention was not to manipulate familiarity directly, but rather to ensure attention to the stimuli while minimizing task-related decision-making, explicit memory retrieval demands, or strategic processing that could confound familiarity-related neural responses. Familiar and unfamiliar faces were viewed under identical task conditions, allowing familiarity-related effects to be examined in the absence of explicit familiarity judgments. Thus, the observed familiarity-related neural dynamics cannot be attributed to differences in task instructions or retrieval requirements. Importantly, these familiarity effects emerged despite the minimal task demands, suggesting that familiarity modulates neural synchronization and network dynamics through processes that are automatically engaged during familiar face viewing, rather than being driven by explicit recognition decisions. Together, our findings indicate that familiarity-related neural dynamics can be observed even under passive viewing conditions that require only sustained attention to the stimuli.

#### Intracranial electrophysiology

We recorded single-neuron activity using microwires embedded in hybrid depth macro-electrodes (Ad-Tech Behnke-Fried electrodes in 14 patients and DIXI macro-micro electrodes in 2 patients) implanted in the amygdala and hippocampus. Broadband extracellular signals (0.1–9000 Hz) were captured from each microwire and continuously stored for offline analysis using either the Blackrock system (30 kHz sampling rate; WUSTL) or the Neuralynx system (32 kHz sampling rate; WVU).^69^ The raw single-neuron data were filtered with a zero-phase lag bandpass filter (300–3000 Hz), and spikes were sorted using a semi-automatic template-matching algorithm.^70^ We then extracted the mean firing rate for each neuron within a time window of 0.25–1.25 s after stimulus onset for each face image.

We recorded iEEG activity via clinical depth macro-electrodes (Ad-Tech, PMT, or DIXI), each consisting of 4–18 channels. Data were analog-filtered above 0.01 Hz and digitally sampled at 2000 Hz, acquired on a Nihon Kohden recording system. Preprocessing was carried out with EEGLAB^71^ and custom MATLAB code. Channels contaminated by epileptic or artifactual activity were manually excluded. Signals were then high-pass filtered at 0.5 Hz and re-referenced with the small Laplacian method (7 mm distance to a set of surrounding channels),^72^ followed by a notch filter to eliminate line noise at 60 Hz and its harmonics (120 Hz and 180 Hz). High-amplitude noise events and interictal discharges were detected and removed on a trial-by-trial basis using a thresholding procedure described in.^64^

At WVU, single-unit recordings were time-locked to stimuli via TTL pulses from the stimulus computer, while no iEEG was recorded at WVU (Table S1). At WUSTL, stimulus timing was recorded using a photodiode placed on the patient’s screen; the analog photodiode signals were recorded as an additional channel and subsequently parsed into triggers for alignment with neural data.

A general consideration when interpreting intracranial electrophysiological data is that recordings are obtained from patients with drug-resistant epilepsy, in whom seizure history, interictal discharges, and underlying pathology may influence baseline neural dynamics. Although trials containing overt interictal discharges were excluded from all analyses, their occurrence indicates that baseline electrophysiology in some recorded regions may exhibit atypical patterns of synchrony. This limitation is inherent to human intracranial studies and should be considered when evaluating the generalizability of the present findings. Importantly, however, our main results were robust to variation in interictal activity across channels. When analyses were repeated using a subset of VTC and MTL channels with fewer than 5% rejected trials—reflecting comparatively lower interictal activity—we observed qualitatively similar functional connectivity patterns (Figure S5). These findings suggest that the reported effects are not driven by interictal contamination and likely reflect task-related neural interactions rather than pathological synchrony.

#### Anatomical localization of iEEG channels

We performed channel localization using pre-operative high-resolution T1-weighted MRI scans and post-operative CT scans. The CT images were co-registered with the MRI scans for patient-specific channel localization, utilizing the VERA software suite (https://github.com/neurotechcenter/VERA). Each channel was assigned to a specific anatomical area based on FreeSurfer’s automatic segmentation.^73^ Channel coordinates were then normalized to the MNI space through nonlinear co-registration for visualization purpose.

Notably, the current study covered iEEG recordings not only from the posterior regions in the VTC, but also the anterior temporal lobe, regions often under-explored due to significant BOLD signal distortion in fMRI studies.^60,74^ Thus, our study can provide valuable insights into the functional organization of the anterior FG, ITG, and MTL, complementing neuroimaging studies.

### QUANTIFICATION AND STATISTICAL ANALYSIS

#### Overview of theta synchronization

In this study, the term *theta synchronization* refers broadly to temporal coordination between neural signals in the theta frequency range but encompasses distinct measurements at different spatial scales. We used pairwise phase consistency (PPC) to quantify spike-field phase locking, which measures the consistency of the timing of neuronal spikes relative to the phase of local field potentials (LFPs; Figures 1 and 2) or intracranial EEG (iEEG; Figure 6) signals. PPC therefore characterizes the temporal relationship between single-neuron activity and ongoing population oscillations. In contrast, phase-locking value (PLV) was used to quantify phase synchronization between continuous iEEG signals recorded from different brain regions (Figure 4), providing a measure of inter-regional temporal coordination at the population level. Finally, Granger causality was used to assess directed temporal dependencies between regional iEEG signals (Figure 4). These metrics capture complementary aspects of neural dynamics across spatial scales, from local spike-field interactions to long-range inter-regional coordination. Importantly, none of these measures should be interpreted as direct evidence of mechanistic information transfer, causal neural communication, or feedforward signaling. Instead, they quantify statistical relationships in neural timing that may arise from multiple underlying mechanisms, including shared input, common network oscillations, recurrent interactions, or directed neuronal influences. In this study, spike-field synchronization (PPC), inter-regional phase synchronization (PLV), and directed temporal dependence (Granger causality) should be distinguished and interpreted according to the level of neural organization they measure.

The theta-band synchrony observed between FG and MTL structures should be interpreted in light of the distinct physiological origins of theta oscillations across cortex, hippocampus, and amygdala. These oscillations arise from different local generators, and transient phase synchronization likely reflects functional coordination rather than a single homogeneous rhythm. Such coupling may align distributed neural populations during face familiarity processing, facilitating selective information exchange without requiring identical oscillatory dynamics. While our analyses do not establish cellular origins or strict directionality, they reveal structured temporal coordination that differentiates familiar from unfamiliar faces, highlighting a mesoscale mechanism supporting cross-regional integration of perceptual and mnemonic information.

Familiarity-related theta synchronization exhibited broadly similar response profiles in the amygdala and hippocampus, with the exception of stronger functional interactions between the FG and the amygdala revealed by PLV and Granger causality analyses (Figure 4). These findings suggest that theta synchronization associated with face familiarity represents a physiological mechanism broadly shared across the human MTL rather than one specific to either the amygdala or hippocampus. At the same time, our results do not exclude finer functional specialization within the MTL. Future studies with larger participant cohorts, richer behavioral paradigms, and causal or perturbational approaches will be important for resolving potential regional differences in familiarity-related neural computations.

#### Spike-field pairwise phase consistency (PPC)

The strength of spike-LFP and spike-iEEG phase locking was quantified by the PPC, which is unbiased by the number of spikes.^75–77^ The PPC quantifies the average similarity (i.e., in-phaseness) of any pair of two spikes from the same neuron in the LFP/iEEG phase domain. For every frequency *f*, we determined the spike-filed phases by cutting out LFP/iEEG segments of length 3/*f* s (i.e., 3 cycles) centered around each spike. For each spike, the surrounding LFP/iEEG was tapered with a Hanning window, with the window length defined as the number of samples corresponding to three cycles of each frequency, and Fourier transformed, and the phase of the spike was determined by frequency decomposition of the LFP/iEEG. The phase similarity was quantified by the PPC as the average phase difference across all possible pairs of spikes within a time window from 0.25 to 1.25 s after stimulus onset. We averaged the phases from the LFP/iEEG recorded across all electrodes within a brain area, excluding the electrode from which the spike-associated LFP was obtained. Asterisks in figures indicate a significant difference between familiar and unfamiliar faces using a two-tailed paired *t* test. *: *p* < 0.05, **: *p* < 0.01, ***: *p* < 0.001, and ****: *p* < 0.0001.

While the PPC solves the problem of sample-size bias, PPC estimates are highly variable for units with a low number of spikes.^75^ To address this problem, we only included neurons that had at least 20 spikes during epochs of interest, which could reduce the variance of the group average.^75^ In addition, to match trial counts between conditions, we performed a bootstrapping procedure with 1000 runs (100 runs for time-frequency analysis). In each run, we randomly subsampled trials to equalize the number of trials between conditions. We then averaged the results across bootstrap runs for each condition in each spike-LFP/iEEG pair.

PPC analyses in this study were performed at the level of spike-LFP or spike-iEEG pairs, resulting in large sample sizes that can produce strong statistical significance despite relatively small effect sizes. Therefore, the reported familiarity-related synchronization effects should be interpreted as modest but reliable shifts in neural population dynamics. To address this issue, we conducted two control analyses. First, we repeated the analyses by averaging across all spike-LFP (or spike-iEEG) pairs for each neuron. Second, we fit a linear mixed-effects model that included neuron and recording session as random effects. Both analyses replicated our original findings. The consistency of these effects across multiple analytical approaches further supports their robustness. Future studies with larger participant cohorts and more comprehensive hierarchical statistical frameworks will be important for further characterizing their biological significance, effect magnitude, and inter-individual variability.

#### High-gamma power (HGP)

High-gamma power (HGP) was extracted by bandpass filtering the signal between 70 and 170 Hz and applying the Hilbert transform. Data were then segmented into event-related epochs spanning −0.5 to 1.5 s relative to stimulus onset, and mean neural responses for each face image were computed within a time window of 0.1–0.6 s after stimulus onset. Asterisks in figures indicate a significant difference between familiar and unfamiliar faces (*p* < 0.05, two-tailed two-sample *t* test, corrected by false discovery rate [FDR]^37^ for *q* < 0.05).

The amygdala and hippocampus exhibited polyphasic responses and differences in HGP between familiar and unfamiliar faces (Figure 3B; Figure S3D), suggesting that face familiarity engages multiple temporally distinct processes within the MTL. Rather than reflecting a single sustained familiarity signal, these dynamics are consistent with the known roles of the amygdala and hippocampus in sequential and recurrent stages of stimulus processing. Early HGP modulations may index rapid detection of salience, affective relevance, or novelty, processes in which the amygdala is critically involved,^78^ whereas later components may reflect associative memory retrieval, contextual integration, or updating supported by hippocampal circuitry.^52,79^ Because HGP reflects aggregate local population activity rather than a unitary computational operation, polyphasic changes are expected in regions that integrate perceptual, affective, and mnemonic information over time. Together, these findings indicate that MTL contributions to face familiarity are dynamic and temporally structured, supporting a view in which familiarity emerges through successive processing stages rather than as a single static signal.

The temporal dissociation between familiarity-related effects in HGP (Figure 3B; Figure S3D) and theta-band phase synchronization (Figure 1E) provides insight into the multistage nature of face familiarity processing. Differences in HGP emerged early (~100–150 ms) across VTC and MTL regions (Figure 3B; Figure S3D), consistent with rapid, stimulus-driven modulation of local population activity during initial visual and identity processing. In contrast, familiarity-related differences in theta-band phase locking (Figure 1E)—most prominently in the amygdala—appeared later (>400 ms), suggesting the engagement of downstream processes. This delayed theta synchrony likely reflects recurrent or integrative interactions across distributed networks, such as associative memory retrieval and affective evaluation, which unfold on slower timescales than early feedforward encoding. Together, these findings support a hierarchical and temporally structured account of face familiarity, in which early local responses are followed by later network-level coordination mediated by theta-band synchronization.

#### Population representational distance of familiarity

For each session, we estimated representational distance using the Euclidean distance between faces across all channels within each ROI, computed separately for familiar and unfamiliar faces. The familiarity-related geometrical distance was then calculated as the difference between the population distances of familiar and unfamiliar faces. This analysis was conducted using the time course of HGP from each channel, although results were comparable when using average HGP.

#### Visual feature extraction

We used the well-known deep neural network (DNN) implementation based on the VGG-16 convolutional neural network (CNN) architecture^80^ to extract features from each CelebA image. Following the same procedure,^28^ we fine-tuned the top layer of the DNN to confirm that the pre-trained model could accurately discriminate face identities (accuracy ≥95%) and ensure its suitability as a facial feature extractor. Notably, neither the feature extraction nor the construction of feature spaces involved any information from neurons.

#### Axis-based feature coding

To identify iEEG channels exhibiting axis-based feature coding—representing the encoding of a linear combination of visual features—we applied partial least squares (PLS) regression using DNN-extracted facial features. Axis-coding channels were selected from face-responsive channels, as this approach assumes visual feature coding. The PLS method has been shown to effectively model neural responses to DNN features.^81,82^ We used four components for the regression, determined through a 10-fold cross-validation procedure to minimize prediction error. The most preferred tuning axis for each axis-coding channel (i.e., the first PLS component) was derived using the full set of images.

To determine statistical significance, we conducted a permutation test with 1000 iterations to evaluate whether a channel encoded a significant face model. In each iteration, 50% of the faces were randomly selected as the training dataset. A model was constructed using the training data to derive regression coefficients, and responses for the remaining 50% of the faces (i.e., the testing dataset) were predicted. The strength of axis coding was assessed by calculating the Pearson correlation between the predicted and actual responses in the testing dataset. This procedure was repeated for 1000 iterations, and the average correlation coefficient was taken as the observed value. Similarly, a null distribution was estimated by shuffling the data. The observed coefficient was then compared against this null distribution, and a channel was considered to encode a significant face model if its observed coefficient exceeded 95% of the values in the null distribution. This method has proven highly effective in identifying neurons with significant axis-coding models.^30^ Additionally, the correlation coefficient reflected the model’s predictability, which is the strength of axis coding, facilitating comparisons across different channels.

#### Phase-locking value (PLV) analysis

We calculated the phase-locking value (PLV)^83^ to quantify the strength of inter-areal neural synchrony between channels. The PLV measures the degree of phase consistency between two channels, with values ranging from 0 to 1. The PLV for a given channel pair was calculated as:

(Equation 1) $$\text{PLV=}\frac{1}{N}\left|\sum_{n=1}^{N}\exp\left(i\left[\theta_{n,a}-\theta_{n,b}\right]\right)\right|$$

where *θ* indicates the phase of channel *a* and channel *b* in each trial *n*. We computed the PLV across frequencies (2–200 Hz) for each VTC–MTL channel pair. For each trial, we extracted the mean phase within a time window of 0–1.5 s after stimulus onset. To mitigate effects introduced by varying trial counts between channels due to artifact rejection, we randomly sampled 400 trials eight times and calculated the average PLV across repetitions. To determine statistical significance for the PLV analysis (Figure 4A), we estimated a null distribution by randomly shuffling the trial labels of one channel in each channel pair prior to calculating the PLV. This procedure was repeated 1000 times for each channel pair, and an observed PLV that exceeded the 95th percentile of the surrogate data was considered significant. For group statistics, we compared the observed mean PLV with the null distribution across all channel pairs.

We compared channel pairs containing VTC axis-coding versus non-axis-coding channels, as well as VTC and MTL channels containing familiarity-coding versus non-familiarity-coding channels (Figures 4A and 4C). To account for unequal group sizes, we used a repeated subsampling procedure. In each iteration, we randomly sampled, without replacement, from the larger group to match the number of channel pairs in the smaller group and calculated the difference in mean PLV between groups. We repeated this procedure 10000 times to obtain a distribution of between-group differences and compared the observed difference with this difference distribution. A one-sided empirical *p*-value was calculated as the proportion of iterations in which the difference was less than 0 for a hypothesized positive effect or greater than 0 for a hypothesized negative effect. Differences were considered statistically significant at *p* < 0.05.

Time–frequency-resolved PLVs were calculated using a 100-ms sliding window advanced in 50-ms steps (Figures 4B, 4D, and 4E). For each VTC–MTL channel pair, PLV at each frequency was normalized relative to the baseline period from −0.5 to −0.1 s before stimulus onset using a *Z* score transformation. The resulting time–frequency maps were then smoothed by cubic-spline interpolation using MATLAB’s spline function. To compare different groups of channel pairs, we applied the same subsampling procedure with 1000 iterations to obtain an empirical *p*-value at each time–frequency point. We subsequently applied a cluster-extent threshold to the statistical map and retained spatially contiguous clusters containing more than 0.5% of all pixels in the time–frequency map.

Finally, PLVs were averaged across the theta-frequency band (3–8 Hz) and the 0–1 s post-stimulus interval. Group means were compared using the 10000-iteration subsampling procedure described above (Figure 4C). Asterisks in figures indicate a significant difference between familiarity-coding and non-familiarity-coding channel pairs. *: *p* < 0.05.

#### Granger causality analysis

To test the directionality of the VTC–MTL interaction, we calculated spectral Granger causality, which quantifies the prediction error of a signal in the frequency domain by incorporating another time series. The time series for each channel was first low-pass filtered at 85 Hz and downsampled to 250 Hz. Next, we normalized the signal within each trial to eliminate amplitude differences and improve signal stationarity. Before fitting the normalized signal to the multivariate autoregressive model, we determined the model order for each session using the Multivariate Granger Causality (MVGC) Toolbox,^84^ based on the Akaike information criterion (AIC). The Granger causality index was computed for the time window of 0–1.5 s after stimulus onset in both directions (VTC to MTL and MTL to VTC). We then applied the same permutation procedure to determine the significance of Granger Causality at each frequency.

We averaged Granger Causality across the theta-frequency band (3–8 Hz) and the 0–1 s post-stimulus interval and compared the group means using the same resampling procedure (see above). Asterisks in figures indicate a significant difference between familiarity-coding and non-familiarity-coding channel pairs. *: *p* < 0.05.

It is worth noting that Granger causality analysis reveals directed temporal dependencies between the VTC and MTL during face familiarity processing. These results indicate asymmetric temporal relationships between regional signals but should not be interpreted as direct evidence of mechanistic information transfer or causal neural communication.

## Supplementary Material

Supplementary Material

Supplemental information can be found online at https://doi.org/10.1016/j.celrep.2026.118014.

## Figures and Tables

**Figure 1.**
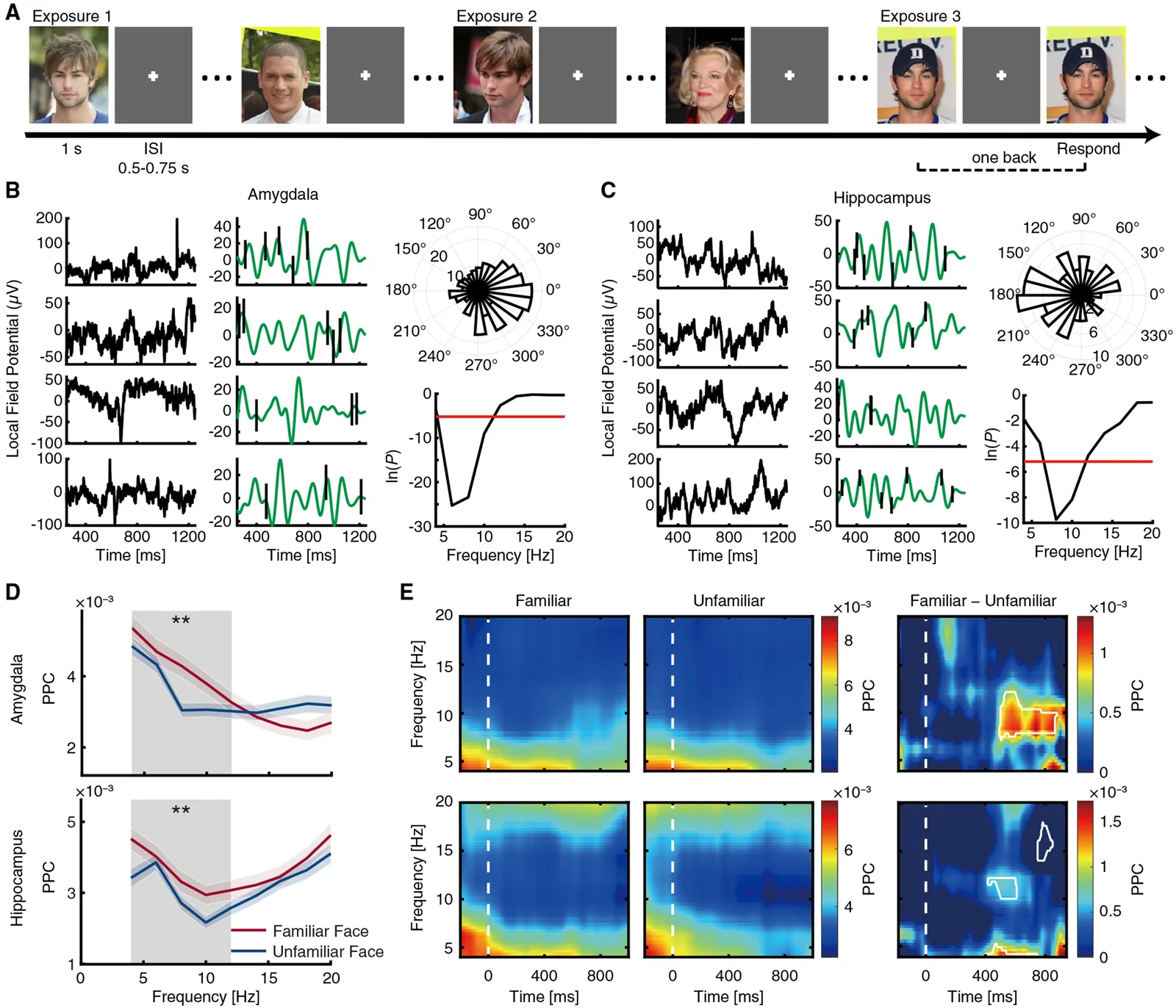
Theta synchronization for face familiarity (A) Task. We employed a one-back task, in which patients responded whenever an identical face was repeated. Each face was presented for 1 s, followed by a jittered inter-stimulus interval (ISI) of 0.5–0.75 s. We showed 10 different images (i.e., 10 exposures) for each of the 50 face identities (totaling 500 unique face images). All face images were presented in random order for each participant. (B) An amygdala neuron. (C) A hippocampal neuron. Left: raw LFP signals. Middle: spikes (vertical lines) on the theta component of the LFP signal. Each row represents a trial segment (250–1,250 ms after stimulus onset). Upper right: distribution of spike phase for the averaged theta oscillation. Lower right: significance of phase locking (Rayleigh test) as a function of frequency (4–20 Hz). The threshold (red line) for significant phase locking was set to *p* = 0.0056 (0.05/9, Bonferroni-corrected). (D) Pairwise phase consistency (PPC) of the spike-LFP pairs (3,712 pairs from 612 neurons for the amygdala [top] and 3,594 pairs from 747 neurons for the hippocampus [bottom]). Error shade denotes ±SEM across spike-LFP pairs (averaged across 1,000 bootstrap runs). The gray shade denotes the theta frequency range (4–12 Hz) for analysis. Asterisks indicate a significant difference between familiar and unfamiliar faces using a two-tailed paired *t* test. **p < 0.01. (E) Time-frequency plots (time window = 1,000 ms, step = 50 ms). Color coding indicates the (difference in) PPC values. White contours mark areas with significant differences between familiar and unfamiliar faces (two-tailed paired *t* test, *p* < 0.05). The white dashed lines represent stimulus onset time. See also Figures S1 and S2 and Table S1.

**Figure 2.**
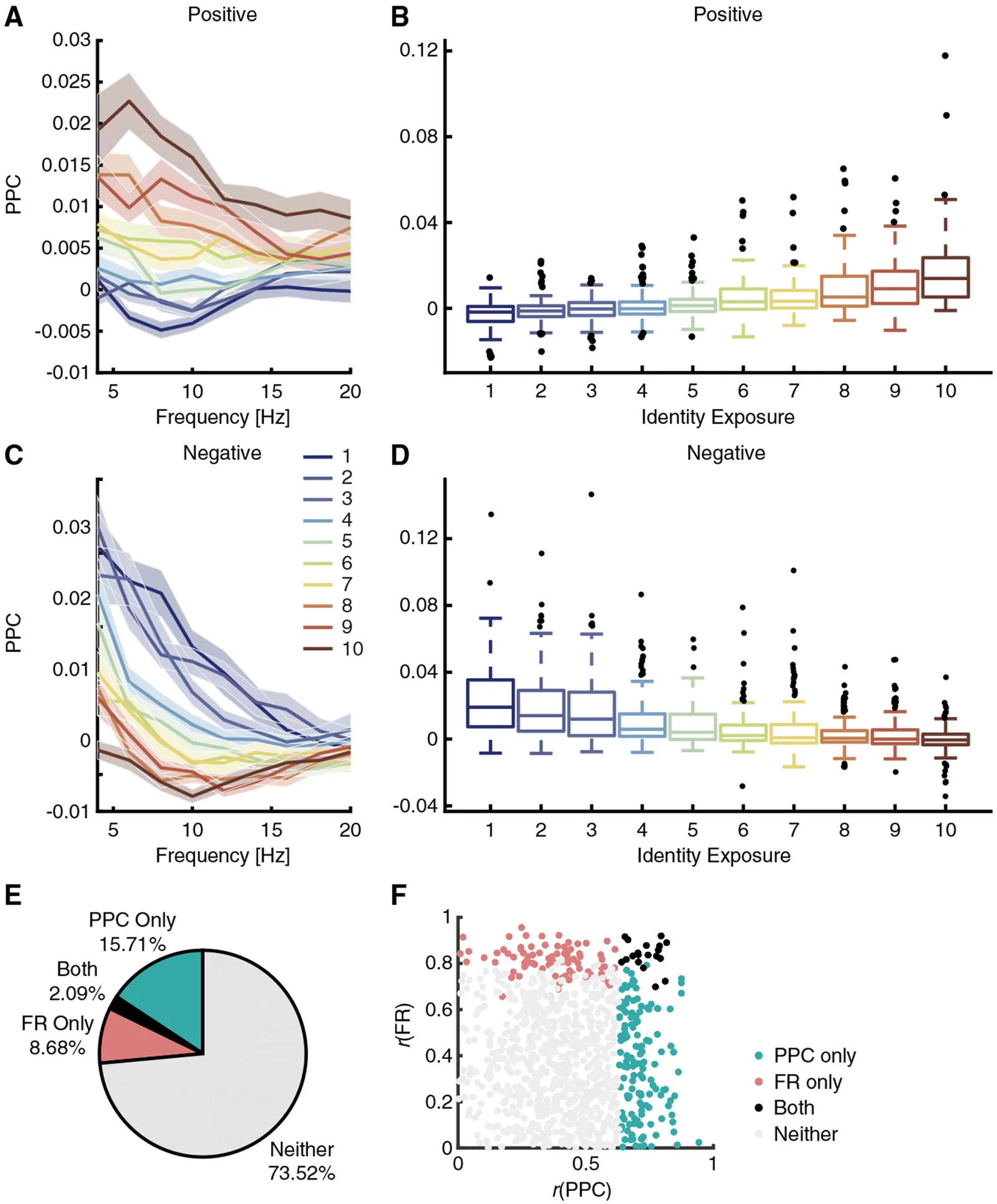
Theta synchronization for face familiarization (A and B) Spike-LFP pairs from theta phase-locking neurons that linearly increased pairwise phase consistency (PPC) with identity exposure (*n* = 104). (C and D) Spike-LFP pairs from theta phase-locking neurons that linearly decreased PPC with identity exposure (*n* = 134). Color coding shows the number of identity exposures. Error shade denotes ±SEM across spike-LFP pairs. For each box, the central mark is the median, the edges of the box are the 25th and 75th percentiles, the whiskers extend to the most extreme data points the algorithm considers to be not outliers, and the outliers are plotted individually. (E and F) Population summary of neurons correlated with identity exposure through theta synchronization (green: PPC only) and/or firing rate (red: FR only; black: both; gray: neither). (F) Correlation coefficients are plotted, with each dot representing a neuron. See also Figures S2

**Figure 3.**
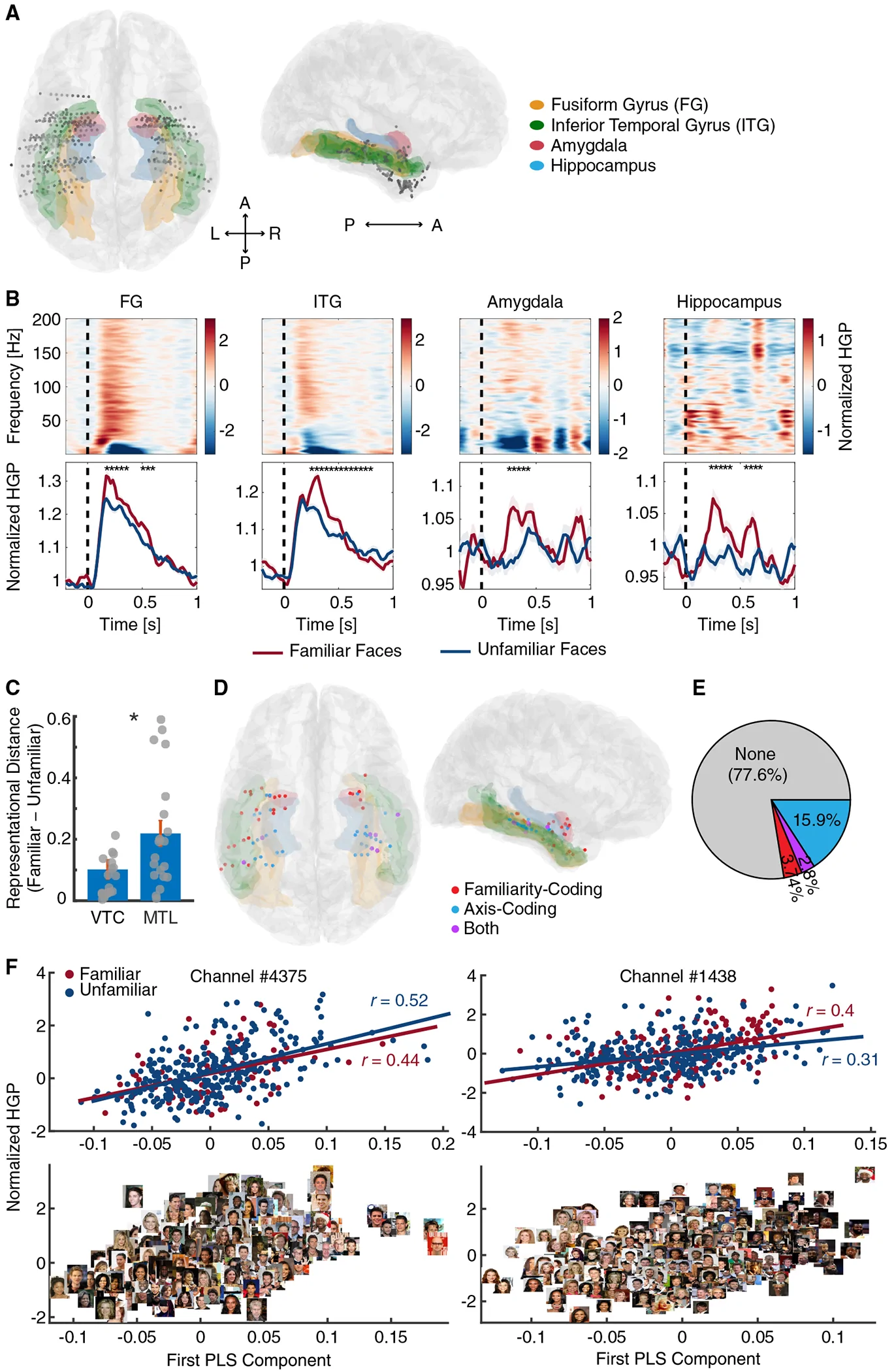
Encoding of familiarity and visual features along the VTC-MTL pathway (A) Distribution of iEEG channels in Montreal Neurological Institute (MNI) space, pooled across participants. Colored regions indicate different anatomical regions of interest (ROIs). L, left; R, right; A, anterior; P, posterior. (B) Representative channels showing significant differences in responses to familiar versus unfamiliar faces. Top: event-related power spectrum averaged across trials. Bottom: time course of high-gamma power (HGP; normalized to pre-trial baseline). Shaded areas indicate ±SEM across trials. Asterisks indicate a significant difference between familiar and unfamiliar faces in that bin (*p* < 0.05, two-tailed two-sample *t* test, corrected by false discovery rate [FDR]^37^ for *q* < 0.05). (C) Population-level neural representation of face familiarity in the VTC and MTL, quantified as the difference in representational distance between familiar and unfamiliar faces. Each dot represents a session, and error bars denote ±SEM across sessions. All channels from each session were included. (D) Spatial distribution of channels showing axis-based feature coding and familiarity encoding, visualized in MNI space. (E) Proportion of visually responsive channels in the VTC exhibiting axis coding and familiarity encoding. (F) Example FG channels showing axis-based feature coding. The responses of both channels exhibited a significant linear relationship with visual features (partial least squares [PLS] regression, permutation test: both *p* values < 0.001). Upper: normalized HGP (*z*-scored) is plotted as a function of the first PLS component (i.e., the most-preferred feature axis). Each dot represents a face image, colored by familiarity. Red and blue lines indicate linear fits for familiar and unfamiliar faces, respectively. The correlation coefficients indicate alignment with the most-preferred feature axis, identified using all images. Lower: visualization of the encoded feature axis. See also Figures S3 and S4 and Table S1.

**Figure 4.**
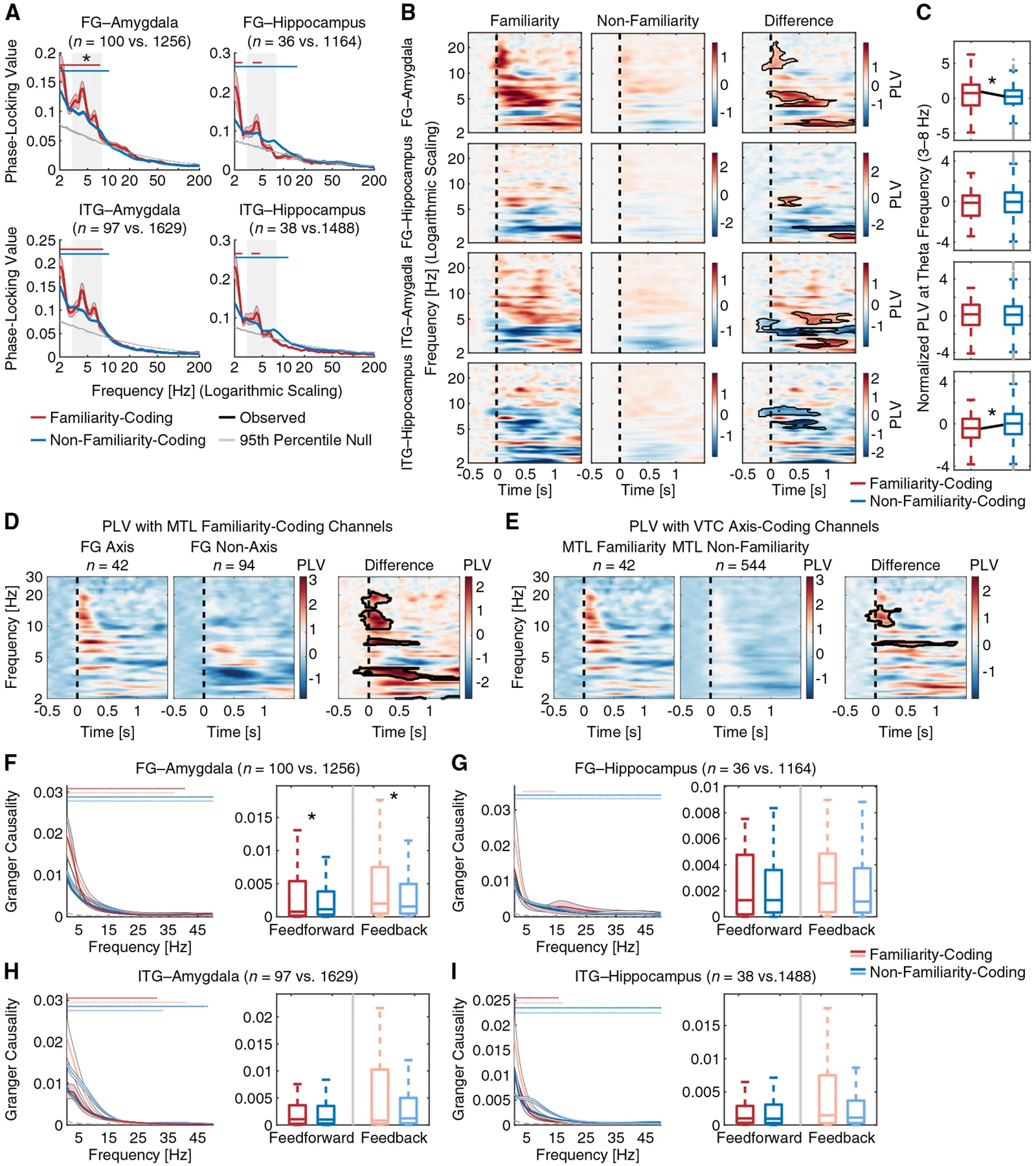
Functional connectivity between the VTC and MTL channels encoding familiarity (A) Phase-locking value (PLV) showing increased VTC-MTL coherence in lower frequencies for both MTL familiarity-coding (red) and non-familiarity-coding (blue) channels. The light gray line indicates the 95th percentile of the null distribution, estimated via permutation (see STAR Methods). The number of channel pairs is indicated. PLVs across channel pairs at each frequency were compared with the 95th percentile of the surrogate distribution. Dots above the line indicate significant PLVs at the corresponding frequencies. Data points with *p* < 0.05 across five consecutive frequencies after FDR correction^37^ were considered significant. The gray-shaded area indicates the theta-frequency range, and asterisks denote a significant difference between familiarity-coding and non-familiarity-coding channel pairs within this range (*p* < 0.05). (B) Time-resolved PLV showing greater synchronization between VTC channels and MTL familiarity-coding channels compared to MTL non-familiarity-coding channels. Each row displays a combination of one MTL subregion (amygdala or hippocampus) and one VTC subregion (FG or ITG). The PLV is normalized to the pre-trial baseline. Contours highlight areas with significant differences, determined by resampling (see STAR Methods). (C) Average PLV within a 0- to 0.8-s window in the theta frequency range (3–8 Hz). Boxplots show the median (central mark), the 25th and 75th percentiles (edges), and whiskers extending to non-outlier extreme values. Outliers are plotted individually. Asterisks indicate a significant difference between familiarity-coding and non-familiarity-coding channel pairs using resampling (10,000 iterations). *p < 0.05. (D) Time-resolved PLV between MTL familiarity-coding channels and VTC axis-coding versus non-axis-coding channels. (E) Time-resolved PLV between VTC axis-coding channels and MTL familiarity-coding versus non-familiarity-coding channels. (F–I) Bidirectional Granger causality (GC) between all combinations of subregions across the VTC and MTL. Left: GC across frequencies. Shaded areas represent ±SEM across channel pairs. The dashed lines indicate the 95th percentile of the null distribution, estimated via permutation. Red: GC between VTC channels and MTL familiarity-coding channels. Blue: GC between VTC channels and MTL non-familiarity-coding channels; dark color: feedforward (from the VTC to the MTL), light color: feedback (from the MTL to the VTC). The dots above indicate significant GC at that frequency (*p* < 0.05 across five consecutive frequencies after FDR correction).^37^ Right: average GC within the theta frequency range (3–8 Hz), separated by feedforward and feedback GC directions. Asterisks indicate a significant difference between familiarity-coding and non-familiarity-coding channel pairs using resampling (10,000 iterations). *p < 0.05. See also Figure S5.

**Figure 5.**
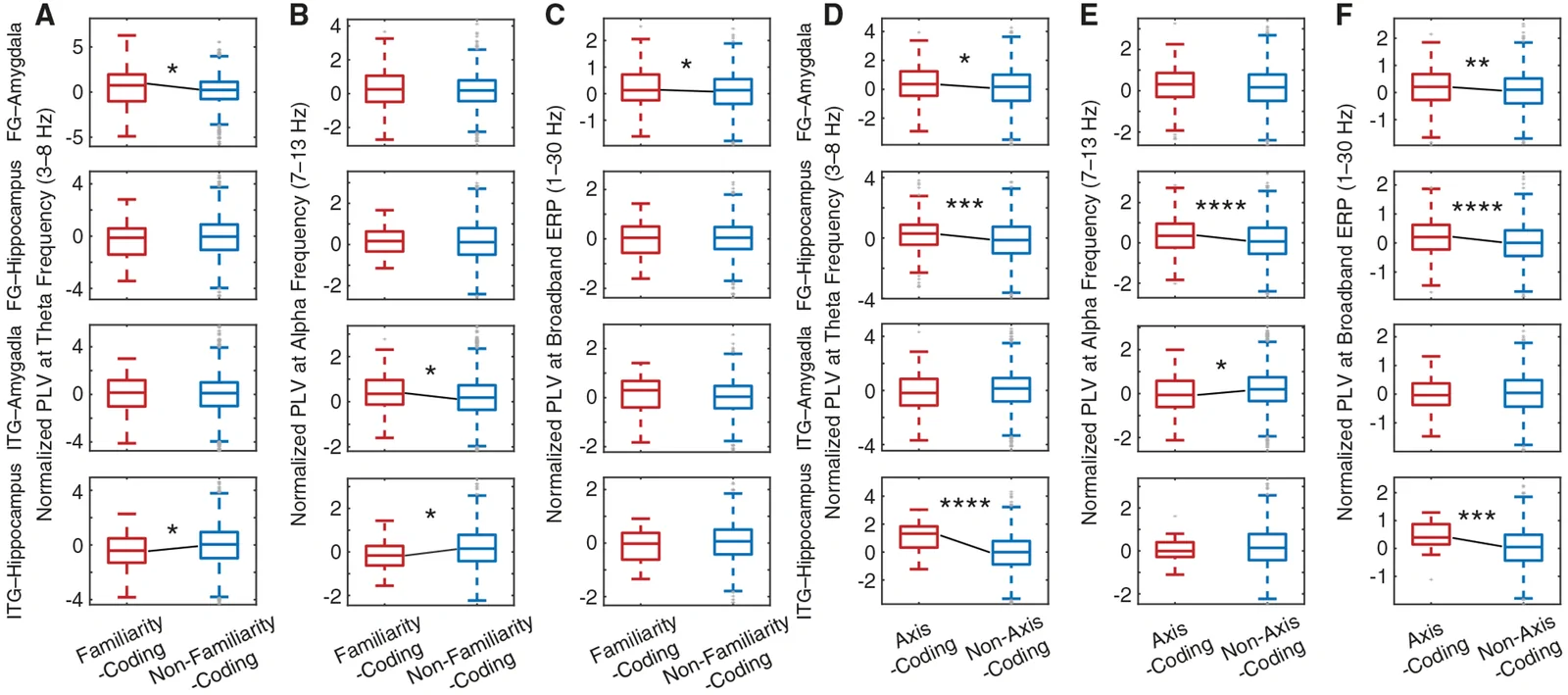
Phase-locking value (PLV) in different frequency bands Average PLV within a 0- to 0.8-s window using (A and D) theta-band, (B and E) alpha-band, and (C and F) broadband event-related potential (ERP) activity. (A–C) Familiarity coding. (D–F) Axis coding. Legend conventions as in Figure 4C. *p < 0.05 , **p < 0.01, ***p < 0.001, and ****p < 0.0001.

**Figure 6.**
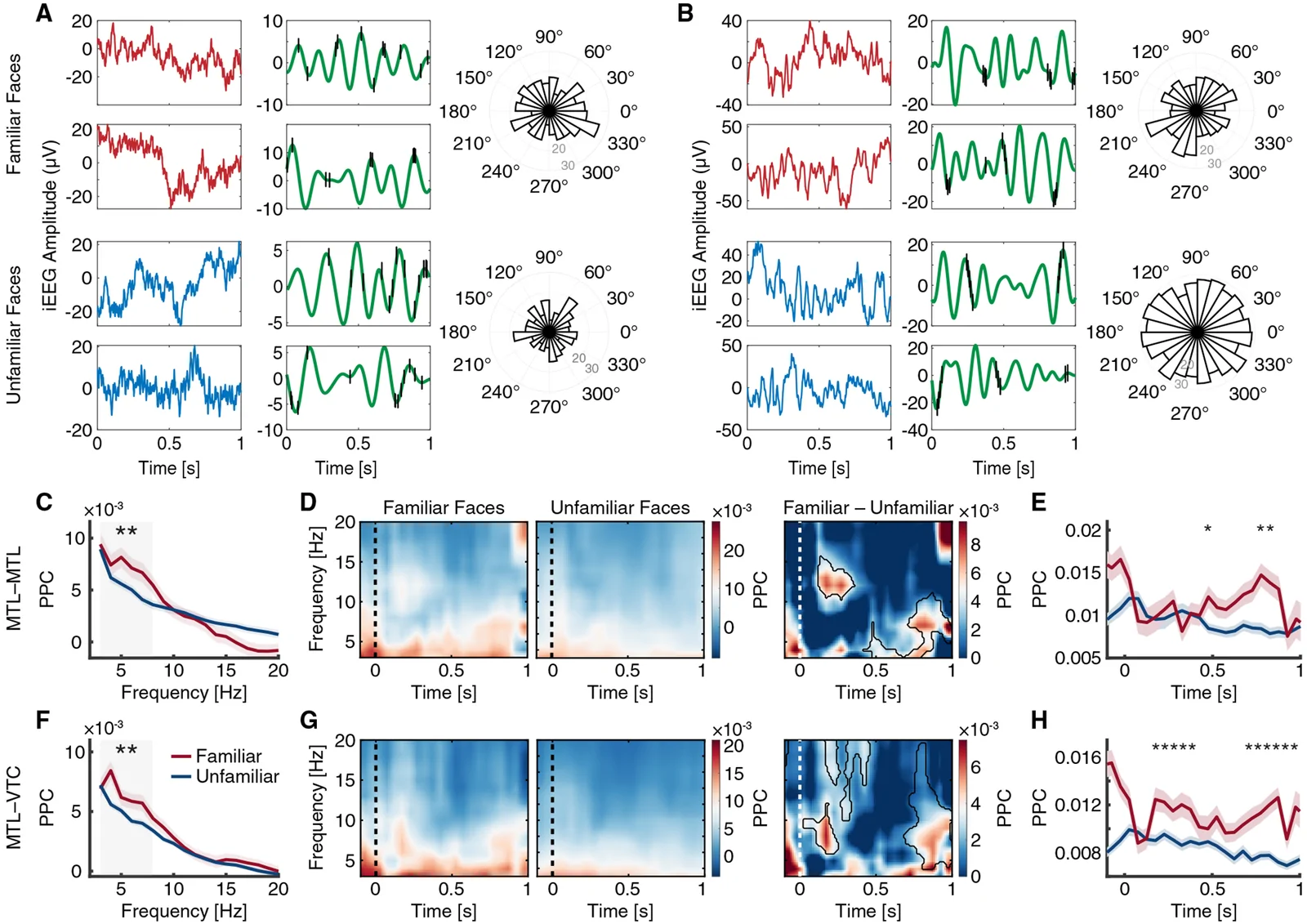
Spike-iEEG coherence for face familiarity in the human temporal lobe (A and B) Example neurons from the medial temporal lobe (MTL) showing phase locking to slow oscillations with iEEG channels within the (A) MTL and (B) ventral temporal cortex (VTC). Left: raw iEEG time series. Middle: spikes (vertical lines) aligned to the theta component of the iEEG. Each row represents a trial segment (0–1,000 ms after stimulus onset). Right: distribution of spike phases for the averaged theta oscillation. The top two rows show trials with familiar faces, and the bottom two rows show trials with unfamiliar faces. (A) Amygdala neuron and amygdala iEEG. (B) Hippocampus neuron and inferior temporal gyrus (ITG) iEEG. (C and F) Pairwise phase consistency (PPC) for the spike-iEEG pairs. Error shade denotes ±SEM across spike-iEEG pairs. The gray shade denotes the theta frequency range (3–8 Hz). Asterisks indicate a significant difference between familiar and unfamiliar faces using a two-tailed paired *t* test. **p < 0.01. (D and G) Time-frequency plots (time window = 500 ms, step = 50 ms). Colors indicate PPC values. Black contours mark areas with significant differences between familiar and unfamiliar faces (one-tailed two-sample *t* test, *p* < 0.05). Vertical dashed lines indicate stimulus onset. (E and H) Mean PPC within the theta frequency range across time. Top bars indicate significant differences between familiar and unfamiliar faces using a two-tailed paired *t* test (*p* < 0.05 across five consecutive frequencies after FDR correction).^37^ Shaded areas indicate ±SEM across spike-iEEG pairs. (C–E) MTL neuron and MTL iEEG (1,536 pairs). (F–H) MTL neuron and VTC iEEG (2,956 pairs). See also Figure S6

**Table:** KEY RESOURCES TABLE

REAGENT or RESOURCE	SOURCE	IDENTIFIER
Deposited data		
Behavioral, single-unit, local field potential, and iEEG data	This paper	Main data/code repository: OSF: https://osf.io/st98x/
Software and algorithms		
MATLAB R2022a	MathWorks	https://www.mathworks.com/products/matlab.html
OSort	N/A	https://www.urut.ch/new/serendipity/index.php?/pages/osort.html
Psychophysics toolbox PTB3	N/A	http://psychtoolbox.org
FieldTrip	N/A	https://www.fieldtriptoolbox.org
Other		
Neuralynx Neurophysiology System	Neuralynx (https://neuralynx.com)	Cat# ATLAS 128
Blackrock Neurophysiology System	Blackrock Microsystems (www.blackrockmicro.com)	Cat# NeuroPort 128
Ad-Tech 8-contact Microelectrode	Ad-Tech (https://adtechmedical.com/subdural-electrodes)	Cat# WB09R-SP00X-014
Ad-Tech Depth sEEG Macroelectrode	Ad-Tech (https://adtechmedical.com/subdural-electrodes)	Cat# BF08R-SP21X-0C2
PMT Depth sEEG Macroelectrode	PMT (https://www.pmtcorp.com/seeg.html)	Cat# 2102–08-IL-SS
DIXI Depth sEEG Macroelectrode	DIXI (https://diximedus.com/products/microdeepseeg-electrodes/)	Cat# MICRODEEP ^®^

## Data Availability

Behavioral, single-unit, local field potential, and intracranial EEG data have been deposited at OSF and are publicly available as of the date of publication. Accession numbers are listed in the key resources table. All original code has been deposited at OSF (https://osf.io/st98x/) and is publicly available as of the date of publication. Any additional information required to reanalyze the data reported in this paper is available from the lead contact upon request.

## Highlights

- Theta phase locking provides an independent neural mechanism for face familiarity
- VTC axis-based visual coding preferentially supports face familiarity
- Familiarity strengthens theta synchronization between the VTC and MTL
- Theta coupling links visual feature processing with memory representations

## References

[1] Bruce V. (1979). Searching for Politicians: An Information-Processing Approach to Face Recognition. Q. J. Exp. Psychol. 31, 373–396. 10.1080/14640747908400734.

[2] Ellis HD, Shepherd JW, and Davies GM. (1979). Identification of familiar and unfamiliar faces from internal and external features: some implications for theories of face recognition. Perception 8, 431–439. 10.1068/p080431. 503774

[3] Kramer RSS, Young AW, and Burton AM. (2018). Understanding face familiarity. Cognition 172, 46–58. 10.1016/j.cognition.2017.12.005. 29232594

[4] Young AW, Reid I, Wright S, and Hellawell DJ. (1993). Face-processing impairments and the Capgras delusion. Br. J. Psychiatry 162, 695–698. 10.1192/bjp.162.5.695. 8149127

[5] Barton JJS. (2003). Disorders of face perception and recognition. Neurol. Clin. 21, 521–548. 10.1016/s0733-8619(02)00106-8. 12916490

[6] Atkinson AP, and Adolphs R. (2011). The neuropsychology of face perception: beyond simple dissociations and functional selectivity. Philos. Trans. R. Soc. B 366, 1726–1738. 10.1098/rstb.2010.0349. PMC3130374 21536556

[7] Wiese H, Schweinberger SR, and Kovács G. (2024). The neural dynamics of familiar face recognition. Neurosci. Biobehav. Rev. 167, 105943. 10.1016/j.neubiorev.2024.105943. 39557351

[8] Bruce V. (1982). Changing faces: visual and non-visual coding processes in face recognition. Br. J. Psychol. 73, 105–116. 10.1111/j.2044-8295.1982.tb01795.x. 7059746

[9] Davies G, and Milne A. (1982). Recognizing Faces in and out of Context. Curr. Psychol. 2, 235–246. 10.1007/BF02684516.

[10] Johnston RA, and Edmonds AJ. (2009). Familiar and unfamiliar face recognition: a review. Memory 17, 577–596. 10.1080/09658210902976969. 19548173

[11] Ramon M, and Gobbini MI. (2018). Familiarity matters: A review on prioritized processing of personally familiar faces. Vis. cogn. 26, 179–195. 10.1080/13506285.2017.1405134.

[12] Kovács G. (2020). Getting to Know Someone: Familiarity, Person Recognition, and Identification in the Human Brain. J. Cognit. Neurosci. 32, 2205–2225. 10.1162/jocn_a_01627. 32902334

[13] Haxby JV, Hoffman EA, and Gobbini MI. (2000). The distributed human neural system for face perception. Trends Cognit. Sci. 4, 223–233. 10.1016/s1364-6613(00)01482-0. 10827445

[14] Duchaine B, and Yovel G. (2015). A Revised Neural Framework for Face Processing. Annu. Rev. Vision Sci. 1, 393–416. 10.1146/annurev-vision-082114-035518. 28532371

[15] Ramon M, Vizioli L, Liu-Shuang J, and Rossion B. (2015). Neural microgenesis of personally familiar face recognition. Proc. Natl. Acad. Sci. USA 112, E4835–E4844. 10.1073/pnas.1414929112. PMC4568242 26283361

[16] Visconti di Oleggio Castello M, Halchenko YO, Guntupalli JS, Gors JD, and Gobbini MI. (2017). The neural representation of personally familiar and unfamiliar faces in the distributed system for face perception. Sci. Rep. 7, 12237, ARTN 12237. 10.1038/s41598-017-12559-1. PMC5612994 28947835

[17] Noad KN, Watson DM, and Andrews TJ. (2025). Conceptual Knowledge Shapes the Neural Representations of Learned Faces in Non-Visual Regions of the Brain. J. Neurosci. 45, e0122252025. 10.1523/JNEUROSCI.0122-25.2025. PMC12311759 40623840

[18] Cao R, Wang J, Brunner P, Willie JT, Li X, Rutishauser U, Brandmeir NJ, and Wang S. (2024). Neural mechanisms of face familiarity and learning in the human amygdala and hippocampus. Cell Rep. 43, 113520. 10.1016/j.celrep.2023.113520. PMC10834150 38151023

[19] Kosaka H, Omori M, Iidaka T, Murata T, Shimoyama T, Okada T, Sadato N, Yonekura Y, and Wada Y. (2003). Neural substrates participating in acquisition of facial familiarity: an fMRI study. Neuroimage 20, 1734–1742. 10.1016/s1053-8119(03)00447-6. 14642483

[20] Leube DT, Erb M, Grodd W, Bartels M, and Kircher TTJ. (2003). Successful episodic memory retrieval of newly learned faces activates a left fronto-parietal network. Cogn. Brain Res. 18, 97–101. 10.1016/j.cogbrainres.2003.09.008. 14659501

[21] Gobbini MI, and Haxby JV. (2006). Neural response to the visual familiarity of faces. Brain Res. Bull. 71, 76–82. 10.1016/j.brainresbull.2006.08.003. 17113931

[22] Nielson KA, Seidenberg M, Woodard JL, Durgerian S, Zhang Q, Gross WL, Gander A, Guidotti LM, Antuono P, and Rao SM. (2010). Common neural systems associated with the recognition of famous faces and names: an event-related fMRI study. Brain Cognit. 72, 491–498. 10.1016/j.bandc.2010.01.006. PMC2935638 20167415

[23] Natu V, and O’Toole AJ. (2011). The neural processing of familiar and unfamiliar faces: a review and synopsis. Br. J. Psychol. 102, 726–747. 10.1111/j.2044-8295.2011.02053.x. 21988381

[24] Quian Quiroga R, Boscaglia M, Jonas J, Rey HG, Yan X, Maillard L, Colnat-Coulbois S, Koessler L, and Rossion B. (2023). Single neuron responses underlying face recognition in the human midfusiform face-selective cortex. Nat. Commun. 14, 5661. 10.1038/s41467-023-41323-5. PMC10499913 37704636

[25] Natu VS, Jiang F, Narvekar A, Keshvari S, Blanz V, and O’Toole AJ. (2010). Dissociable neural patterns of facial identity across changes in viewpoint. J. Cognit. Neurosci. 22, 1570–1582. 10.1162/jocn.2009.21312. 19642884

[26] Goesaert E, and Op de Beeck HP. (2013). Representations of Facial Identity Information in the Ventral Visual Stream Investigated with Multivoxel Pattern Analyses. J. Neurosci. 33, 8549–8558. 10.1523/JNEUROSCI.1829-12.2013. PMC6619647 23658192

[27] Cao R, He S, and Zhang P. (2015). Identity-specific adaptation to invisible faces depends on the depth of interocular suppression. J. Vis. 15, 383. 10.1167/15.12.383.

[28] Cao R, Wang J, Lin C, De Falco E, Peter A, Rey HG, Brunner P, Willie JT, DiCarlo JJ, Todorov A, (2025). Feature-based encoding of face identity by single neurons in the human amygdala and hippocampus. Nat. Hum. Behav. 9, 1959–1974. 10.1038/s41562-025-02218-1. PMC12240612 40481217

[29] Cao R, Zhang J, Zheng J, Wang Y, Brunner P, Willie JT, and Wang S. (2025). A neural computational framework for face processing in the human temporal lobe. Curr. Biol. 35, 1765–1778.e6. 10.1016/j.cub.2025.02.063. PMC12014353 40118061

[30] Chang L, and Tsao DY. (2017). The Code for Facial Identity in the Primate Brain. Cell 169, 1013–1028.e14. 10.1016/j.cell.2017.05.011. PMC8088389 28575666

[31] Quiroga RQ, Reddy L, Kreiman G, Koch C, and Fried I. (2005). Invariant visual representation by single neurons in the human brain. Nature 435, 1102–1107. 10.1038/nature03687. https://www.nature.com/nature/journal/v435/n7045/suppinfo/nature03687_S1.html. 15973409

[32] Quiroga RQ. (2012). Concept cells: the building blocks of declarative memory functions. Nat. Rev. Neurosci. 13, 587–597. 10.1038/nrn3251. 22760181

[33] Cao R, Brunner P, Chakravarthula PN, Wahlstrom KL, Inman C, Smith EH, Li X, Mamelak AN, Brandmeir NJ, Rutishauser U, (2025). A neuronal code for object representation and memory in the human amygdala and hippocampus. Nat. Commun. 16, 1510. 10.1038/s41467-025-56793-y. PMC11811184 39929825

[34] She L, Benna MK, Shi Y, Fusi S, and Tsao DY. (2024). Temporal multiplexing of perception and memory codes in IT cortex. Nature 629, 861–868. 10.1038/s41586-024-07349-5. PMC11111405 38750353

[35] Viskontas IV, Quiroga RQ, and Fried I. (2009). Human medial temporal lobe neurons respond preferentially to personally relevant images. Proc. Natl. Acad. Sci. USA 106, 21329–21334. 10.1073/pnas.0902319106. PMC2795562 19955441

[36] Donoghue T, Haller M, Peterson EJ, Varma P, Sebastian P, Gao R, Noto T, Lara AH, Wallis JD, Knight RT, (2020). Parameterizing neural power spectra into periodic and aperiodic components. Nat. Neurosci. 23, 1655–1665. 10.1038/s41593-020-00744-x. PMC8106550 33230329

[37] Benjamini Y, and Hochberg Y. (1995). Controlling the False Discovery Rate: A Practical and Powerful Approach to Multiple Testing. J. Roy. Stat. Soc. B Stat. Methodol. 57, 289–300. 10.1111/j.2517-6161.1995.tb02031.x.

[38] Ray S, Crone NE, Niebur E, Franaszczuk PJ, and Hsiao SS. (2008). Neural correlates of high-gamma oscillations (60-200 Hz) in macaque local field potentials and their potential implications in electrocorticography. J. Neurosci. 28, 11526–11536. 10.1523/JNEUROSCI.2848-08.2008. PMC2715840 18987189

[39] Lachaux JP, Axmacher N, Mormann F, Halgren E, and Crone NE. (2012). High-frequency neural activity and human cognition: past, present and possible future of intracranial EEG research. Prog. Neurobiol. 98, 279–301. 10.1016/j.pneurobio.2012.06.008. PMC3980670 22750156

[40] Parvizi J, and Kastner S. (2018). Promises and limitations of human intracranial electroencephalography. Nat. Neurosci. 21, 474–483. 10.1038/s41593-018-0108-2. PMC6476542 29507407

[41] Fairhall SL, and Ishai A. (2007). Effective connectivity within the distributed cortical network for face perception. Cerebr. Cortex 17, 2400–2406. 10.1093/cercor/bhl148. 17190969

[42] Wang Y, Metoki A, Smith DV, Medaglia JD, Zang Y, Benear S, Popal H, Lin Y, and Olson IR. (2020). Multimodal mapping of the face connectome. Nat. Hum. Behav. 4, 397–411. 10.1038/s41562-019-0811-3. PMC7167350 31988441

[43] Kessler R, Rusch KM, Wende KC, Schuster V, and Jansen A. (2021). Revisiting the effective connectivity within the distributed cortical network for face perception. Neuroimage: Reports 1, 100045. 10.1016/j.ynirp.2021.100045. PMC12172785 40568430

[44] Sato W, Kochiyama T, Uono S, Matsuda K, Usui K, Inoue Y, and Toichi M. (2014). Rapid, high-frequency, and theta-coupled gamma oscillations in the inferior occipital gyrus during face processing. Cortex 60, 52–68. 10.1016/j.cortex.2014.02.024. 24745564

[45] Rutishauser U, Ross IB, Mamelak AN, and Schuman EM. (2010). Human memory strength is predicted by theta-frequency phase-locking of single neurons. Nature 464, 903–907. 10.1038/nature08860. 20336071

[46] Herweg NA, Solomon EA, and Kahana MJ. (2020). Theta Oscillations in Human Memory. Trends Cognit. Sci. 24, 208–227. 10.1016/j.tics.2019.12.006. PMC8310425 32029359

[47] Buzsáki G. (2002). Theta Oscillations in the Hippocampus. Neuron 33, 325–340. 10.1016/S0896-6273(02)00586-X. 11832222

[48] Varela F, Lachaux JP, Rodriguez E, and Martinerie J. (2001). The brainweb: phase synchronization and large-scale integration. Nat. Rev. Neurosci. 2, 229–239. 10.1038/35067550. 11283746

[49] Fries P. (2005). A mechanism for cognitive dynamics: neuronal communication through neuronal coherence. Trends Cognit. Sci. 9, 474–480. 10.1016/j.tics.2005.08.011. 16150631

[50] Roelfsema PR. (2023). Solving the binding problem: Assemblies form when neurons enhance their firing rate-they don’t need to oscillate or synchronize. Neuron 111, 1003–1019. 10.1016/j.neuron.2023.03.016. 37023707

[51] Vinck M, Uran C, Spyropoulos G, Onorato I, Broggini AC, Schneider M, and Canales-Johnson A. (2023). Principles of large-scale neural interactions. Neuron 111, 987–1002. 10.1016/j.neuron.2023.03.015. 37023720

[52] Squire LR, Stark CEL, and Clark RE. (2004). The Medial Temporal Lobe. Annu. Rev. Neurosci. 27, 279–306. 10.1146/annurev.neuro.27.070203.144130. 15217334

[53] Li J, Liu J, Liang J, Zhang H, Zhao J, Rieth CA, Huber DE, Li W, Shi G, Ai L, (2010). Effective connectivities of cortical regions for top-down face processing: a dynamic causal modeling study. Brain Res. 1340, 40–51. 10.1016/j.brainres.2010.04.044. PMC3724518 20423709

[54] Herrington JD, Taylor JM, Grupe DW, Curby KM, and Schultz RT. (2011). Bidirectional communication between amygdala and fusiform gyrus during facial recognition. Neuroimage 56, 2348–2355. 10.1016/j.neuroimage.2011.03.072. PMC3137553 21497657

[55] Wang X, Zhen Z, Song Y, Huang L, Kong X, and Liu J. (2016). The Hierarchical Structure of the Face Network Revealed by Its Functional Connectivity Pattern. J. Neurosci. 36, 890–900. 10.1523/JNEUROSCI.2789-15.2016. PMC6601995 26791218

[56] Noad KN, Watson DM, and Andrews TJ. (2024). Familiarity enhances functional connectivity between visual and nonvisual regions of the brain during natural viewing. Cerebr. Cortex 34, bhae285, ARTN bhae285. 10.1093/cercor/bhae285. 39038830

[57] Lohmann G, Erfurth K, Müller K, and Turner R. (2012). Critical comments on dynamic causal modelling. Neuroimage 59, 2322–2329. 10.1016/j.neuroimage.2011.09.025. 22001162

[58] Lohmann G, Müller K, and Turner R. (2013). Response to commentaries on our paper: Critical comments on dynamic causal modelling. Neuroimage 75, 279–281. 10.1016/j.neuroimage.2012.07.041. 29768908

[59] Ojemann JG, Akbudak E, Snyder AZ, McKinstry RC, Raichle ME, and Conturo TE. (1997). Anatomic localization and quantitative analysis of gradient refocused echo-planar fMRI susceptibility artifacts. Neuroimage 6, 156–167. 10.1006/nimg.1997.0289. 9344820

[60] Rossion B, Jacques C, and Jonas J. (2024). The anterior fusiform gyrus: The ghost in the cortical face machine. Neurosci. Biobehav. Rev. 158, 105535. 10.1016/j.neubiorev.2024.105535. 38191080

[61] Mercier MR, Dubarry AS, Tadel F, Avanzini P, Axmacher N, Cellier D, Vecchio MD, Hamilton LS, Hermes D, Kahana MJ, (2022). Advances in human intracranial electroencephalography research, guidelines and good practices. Neuroimage 260, 119438. 10.1016/j.neuroimage.2022.119438. PMC10190110 35792291

[62] Klopp J, Marinkovic K, Chauvel P, Nenov V, and Halgren E. (2000). Early widespread cortical distribution of coherent fusiform face selective activity. Hum. Brain Mapp. 11, 286–293. 10.1002/1097-0193(200012)11:4<286::AID-HBM80>3.0.CO;2-R. PMC6871879 11144757

[63] Zheng J, Anderson KL, Leal SL, Shestyuk A, Gulsen G, Mnatsakanyan L, Vadera S, Hsu FPK, Yassa MA, Knight RT, and Lin JJ. (2017). Amygdala-hippocampal dynamics during salient information processing. Nat. Commun. 8, 14413. 10.1038/ncomms14413. https://www.nature.com/articles/ncomms14413#supplementary-information. PMC5309795 28176756

[64] Daume J, Kamińki J, Schjetnan AGP, Salimpour Y, Khan U, Kyzar M., Reed CM, Anderson WS, Valiante TA, Mamelak AN, and Rutishauser U. (2024). Control of working memory by phase–amplitude coupling of human hippocampal neurons. Nature 629, 393–401. 10.1038/s41586-024-07309-z. PMC11078732 38632400

[65] Cao R, Lin C, Brandmeir NJ, and Wang S. (2022). A human single-neuron dataset for face perception. Sci. Data 9, 365. 10.1038/s41597-022-01482-4. PMC9233707 35752635

[66] Cao R, Zhang J, Zheng J, Wang Y, Brunner P, Willie JT, and Wang S. (2026). Computational single-neuron mechanisms of visual object coding in the human temporal lobe. Nat. Commun. 17, 2234. 10.1038/s41467-026-68954-8. PMC12963616 41620419

[67] Liu Z, Luo P, Wang X, and Tang X. (2015). Deep Learning Face Attributes in the Wild. Proceedings of the IEEE international conference on computer vision (ICCV), 3730–3738. 10.1109/ICCV.2015.425.

[68] Cao R, Li X, Brandmeir NJ, and Wang S. (2021). Encoding of facial features by single neurons in the human amygdala and hippocampus. Commun. Biol. 4, 1394. 10.1038/s42003-021-02917-1. PMC8671411 34907323

[69] Schalk G, McFarland DJ, Hinterberger T, Birbaumer N, and Wolpaw JR. (2004). BCI2000: a general-purpose brain-computer interface (BCI) system. IEEE Trans. Biomed. Eng. 51, 1034–1043. 10.1109/TBME.2004.827072. 15188875

[70] Rutishauser U, Schuman EM, and Mamelak AN. (2006). Online detection and sorting of extracellularly recorded action potentials in human medial temporal lobe recordings, in vivo. J. Neurosci. Methods 154, 204–224. 10.1016/j.jneumeth.2005.12.033. 16488479

[71] Delorme A, and Makeig S. (2004). EEGLAB: an open source toolbox for analysis of single-trial EEG dynamics including independent component analysis. J. Neurosci. Methods 134, 9–21. 10.1016/j.jneumeth.2003.10.009. 15102499

[72] McFarland DJ, McCane LM, David SV, and Wolpaw JR. (1997). Spatial filter selection for EEG-based communication. Electroencephalogr. Clin. Neurophysiol. 103, 386–394. 10.1016/s0013-4694(97)00022-2. 9305287

[73] Fischl B, Salat DH, Busa E, Albert M, Dieterich M, Haselgrove C, van der Kouwe A, Killiany R, Kennedy D, Klaveness S, (2002). Whole brain segmentation: automated labeling of neuroanatomical structures in the human brain. Neuron 33, 341–355. 10.1016/s0896-6273(02)00569-x. 11832223

[74] Devlin JT, Russell RP, Davis MH, Price CJ, Wilson J, Moss HE, Matthews PM, and Tyler LK. (2000). Susceptibility-induced loss of signal: comparing PET and fMRI on a semantic task. Neuroimage 11, 589–600. 10.1006/nimg.2000.0595. 10860788

[75] Vinck M, van Wingerden M, Womelsdorf T, Fries P, and Pennartz CMA. (2010). The pairwise phase consistency: A bias-free measure of rhythmic neuronal synchronization. Neuroimage 51, 112–122. 10.1016/j.neuroimage.2010.01.073. 20114076

[76] Vinck M, Battaglia FP, Womelsdorf T, and Pennartz C. (2012). Improved measures of phase-coupling between spikes and the Local Field Potential. J. Comput. Neurosci. 33, 53–75. 10.1007/s10827-011-0374-4. PMC3394239 22187161

[77] Vinck M, Womelsdorf T, Buffalo EA, Desimone R, and Fries P. (2013). Attentional Modulation of Cell-Class-Specific Gamma-Band Synchronization in Awake Monkey Area V4. Neuron 80, 1077–1089. 10.1016/j.neuron.2013.08.019. PMC3840396 24267656

[78] Wang S and Adolphs R. (2017). Social Saliency. In Computational and Cognitive Neuroscience of Vision, Zhao Q, ed. (Springer Singapore), pp. 171–193. 10.1007/978-981-10-0213-7_8.

[79] Eichenbaum H, Yonelinas AP, and Ranganath C. (2007). The Medial Temporal Lobe and Recognition Memory. Annu. Rev. Neurosci. 30, 123–152. 10.1146/annurev.neuro.30.051606.094328. PMC2064941 17417939

[80] Parkhi OM, Vedaldi A, and Zisserman A. (2015). Deep face recognition, https://www.geeksforgeeks.org/machine-learning/deep-face-recognition/

[81] Yamins DLK, Hong H, Cadieu CF, Solomon EA, Seibert D, and DiCarlo JJ. (2014). Performance-optimized hierarchical models predict neural responses in higher visual cortex. Proc. Natl. Acad. Sci. USA 111, 8619–8624. 10.1073/pnas.1403112111. PMC4060707 24812127

[82] Ponce CR, Xiao W, Schade PF, Hartmann TS, Kreiman G, and Livingstone MS. (2019). Evolving Images for Visual Neurons Using a Deep Generative Network Reveals Coding Principles and Neuronal Preferences. Cell 177, 999–1009.e10. 10.1016/j.cell.2019.04.005. PMC6718199 31051108

[83] Lachaux JP, Rodriguez E, Martinerie J, and Varela FJ. (1999). Measuring phase synchrony in brain signals. Hum. Brain Mapp. 8, 194–208. 10.1002/(sici)1097-0193(1999)8:4<194::aid-hbm4>3.0.co;2-c. PMC6873296 10619414

[84] Barnett L, and Seth AK. (2014). The MVGC multivariate Granger causality toolbox: A new approach to Granger-causal inference. J. Neurosci. Methods 223, 50–68. 10.1016/j.jneumeth.2013.10.018. 24200508

