# Supplementary material for: Theta synchronization coordinates ventral temporal and medial temporal coding of human face familiarity
Source: Cell Rep. Author manuscript; Available in PMC 2026 Oct 14. (PMC13660371; doi:10.1016/j.celrep.2026.118014)
Supplement: Supplementary file 1 [file NIHMS2213046-supplement-Supplementary_Material.pdf]

Cell Reports, Volume *45*

Supplemental information

Theta synchronization coordinates ventral temporal and medial temporal coding of human face familiarity

Yilin Li, Jie Zhang, Yue Wang, Hohyun Cho, Peter Brunner, Jasmine Thum, Jon T. Willie, Runnan Cao, and Shuo Wang

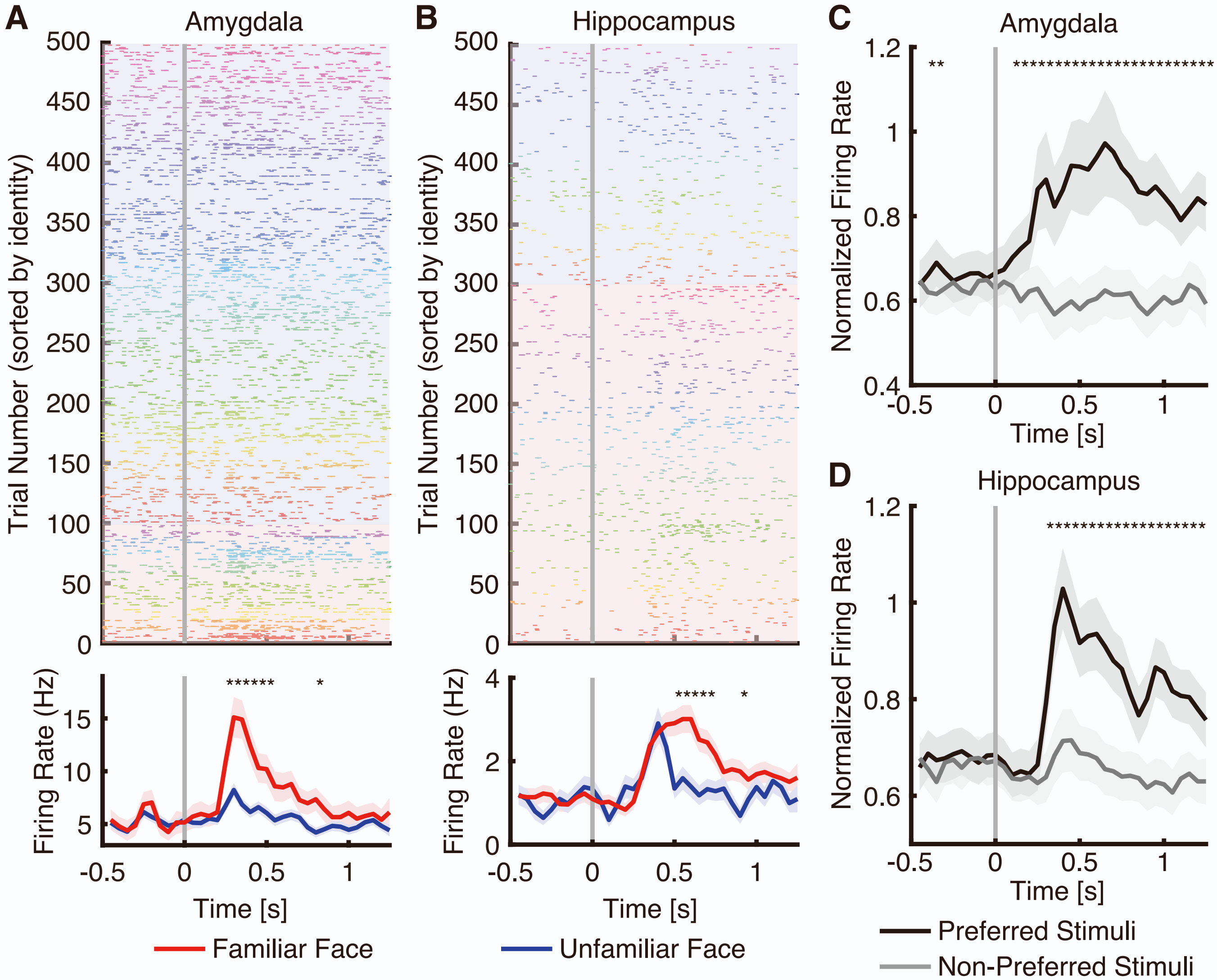

Figure S1. Neurons whose firing rates differentiate familiar versus unfamiliar faces, Related to Figure 1. (A, B) Example neurons that had a greater firing rate for familiar faces (selected by a two-tailed two-sample *t*-test in a time window of 250–1250 ms relative to stimulus onset; both $ps < 0.01$). Trials are aligned to stimulus onset (gray line) and grouped by individual identities (shown by color coding). Shaded areas in the raster plot indicate familiar (red) and unfamiliar (blue) identities. Asterisks indicate a significant difference between familiar versus unfamiliar faces in that bin ($p < 0.05$, two-tailed two-sample *t*-test, corrected by false discovery rate [FDR] for $q < 0.05$; bin size = 50 ms). Note that the selection of neurons was based on the entire time window. **(C, D)** Normalized firing rate as a function of time averaged across all familiarity-selective neurons (i.e., neurons that significantly differentiated familiar versus unfamiliar faces; selected by a two-tailed two-sample *t*-test in a time window of 250–1250 ms relative to stimulus onset). Shaded areas denote ±SEM across neurons. Asterisks indicate a

significant difference between the conditions in that bin ($p < 0.05$, two-tailed paired t-test, corrected by FDR for $Q < 0.05$). Black: preferred stimuli (i.e., familiar faces for familiarity-preferring neurons and unfamiliar faces for unfamiliarity-preferring neurons). Gray: non-preferred stimuli (familiar faces for non-familiarity-preferring stimuli and unfamiliar faces for familiarity-preferring stimuli). **(C)** Amygdala (38 familiarity-preferring and 18 unfamiliarity-preferring). **(D)** Hippocampus (52 familiarity-preferring and 23 unfamiliarity-preferring).

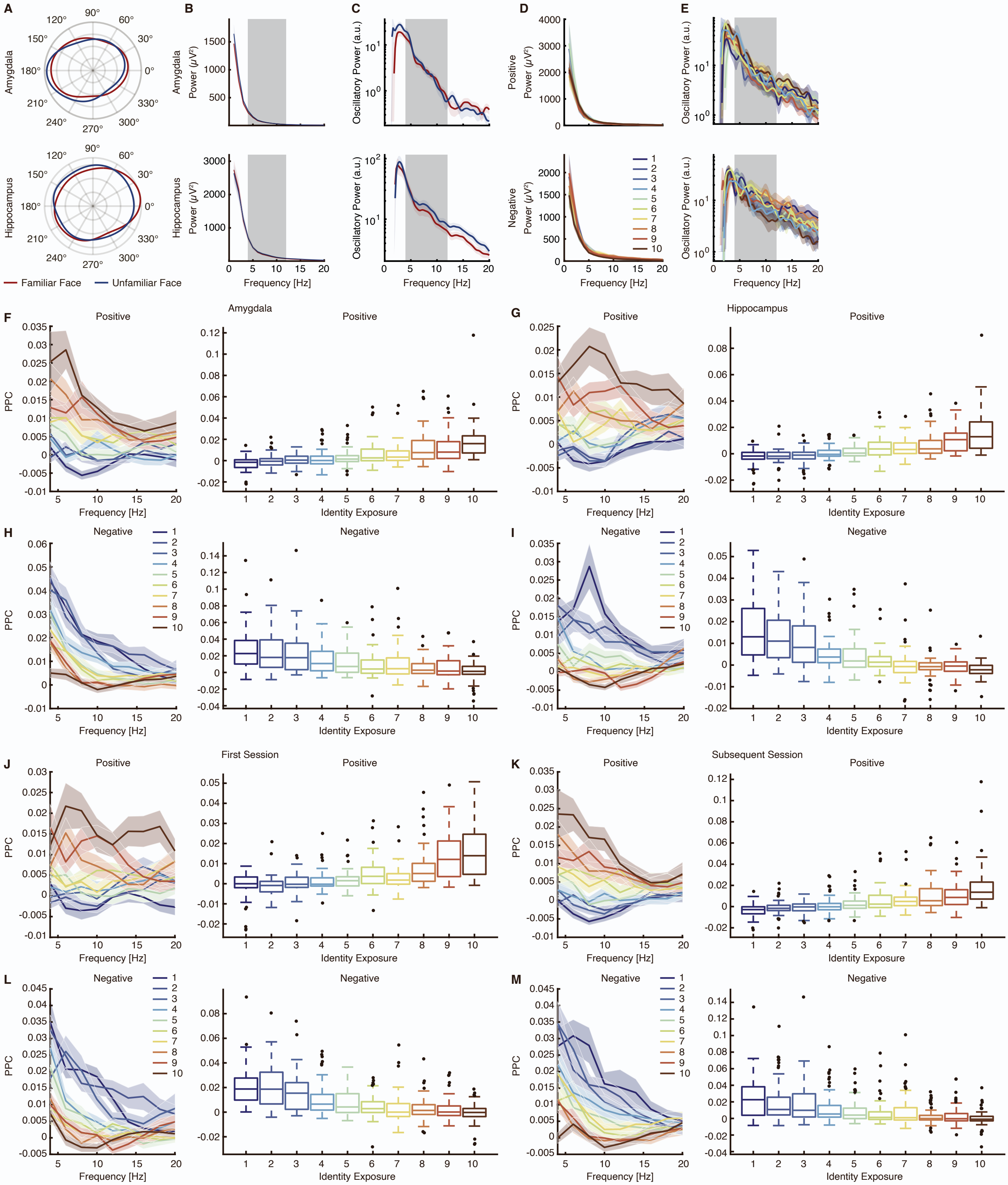

Figure S2. Control analyses of spike–LFP synchronization, Related to Figure 1 and Figure 2. (A) Distribution of spike–LFP phase angles across spike–LFP pairs. **(B, D)** Power spectrum. **(C, E)** Oscillation-specific (periodic) component of the power spectrum. **(B, C)** Face familiarity. **(D, E)** Face

familiarization. **(F–M)** Additional control analyses of theta synchronization during face familiarization. **(F–I)** Separate analysis for the amygdala and hippocampus. **(F, G)** Spike–LFP pairs from theta phase-locking neurons that linearly increased pairwise phase consistency (PPC) with identity exposure (amygdala: n = 52; hippocampus: n = 52). **(H, I)** Spike–LFP pairs from theta phase-locking neurons that linearly decreased PPC with identity exposure (amygdala: n = 76; hippocampus: n = 58). **(F, H)** Amygdala. **(G, I)** Hippocampus. Legend conventions as in **Figure 2**. **(J–M)** Separate analysis for the first and subsequent sessions. **(J, K)** Spike–LFP pairs from theta phase-locking neurons that linearly increased PPC with identity exposure (first session: n = 40; subsequent session: n = 64). **(L, M)** Spike–LFP pairs from theta phase-locking neurons that linearly decreased PPC with identity exposure (first session: n = 61; subsequent session: n = 73). **(J, L)** The first session. **(K, M)** Subsequent sessions.

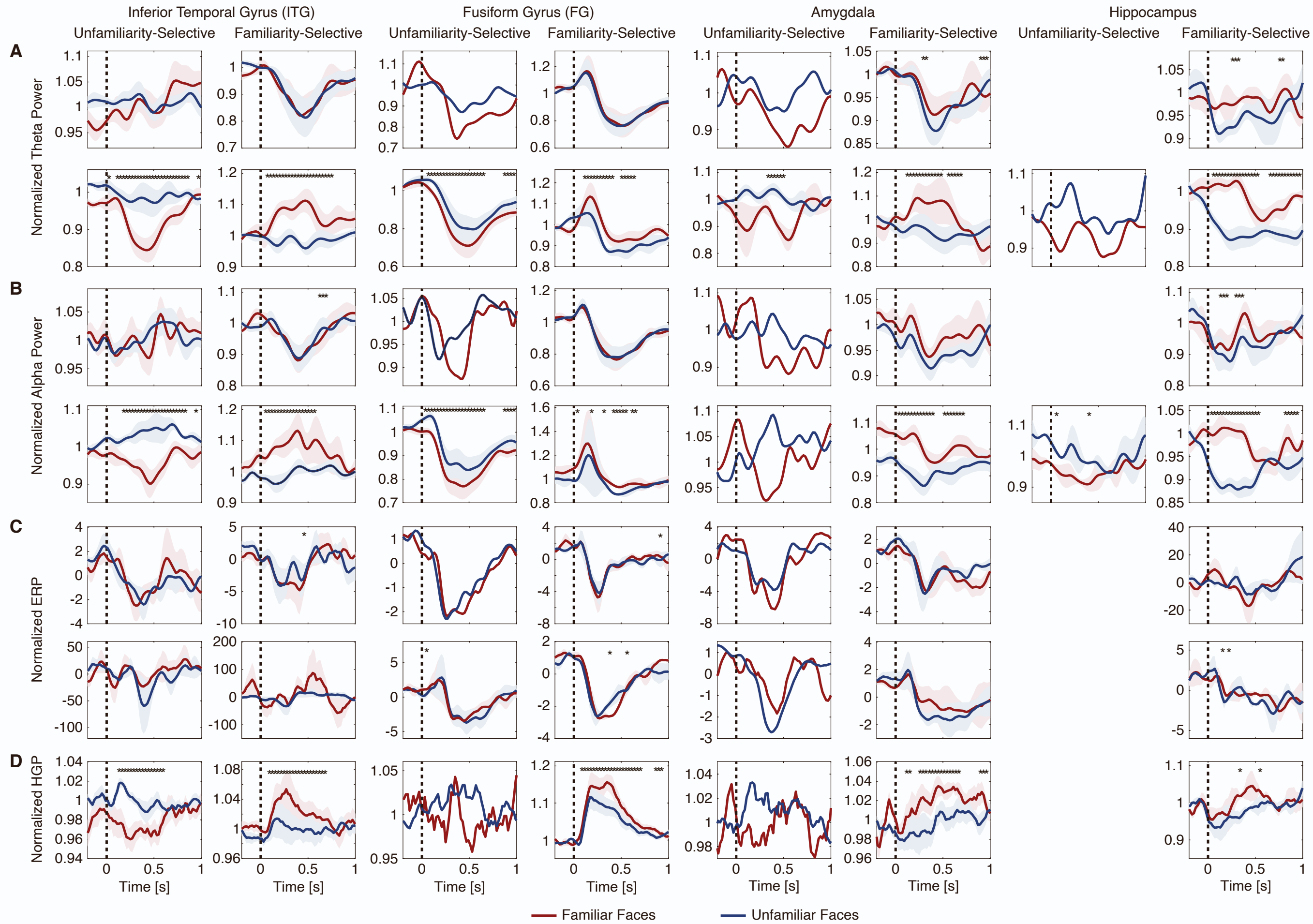

Figure S3. Differences in responses to familiar versus unfamiliar faces, Related to Figure 3. (A) Theta-band power. **(B)** Alpha-band power. **(C)** Broadband event-related potentials (ERPs). (upper) Channels selected based on high-gamma power (HGP). (lower) Channels selected in the corresponding frequency range. **(D)** HGP. Error shades denote ±SEM across channels. Asterisks indicate a significant difference between familiar and unfamiliar faces in that bin ($p < 0.05$, two-tailed paired t-test, corrected by FDR for $q < 0.05$).

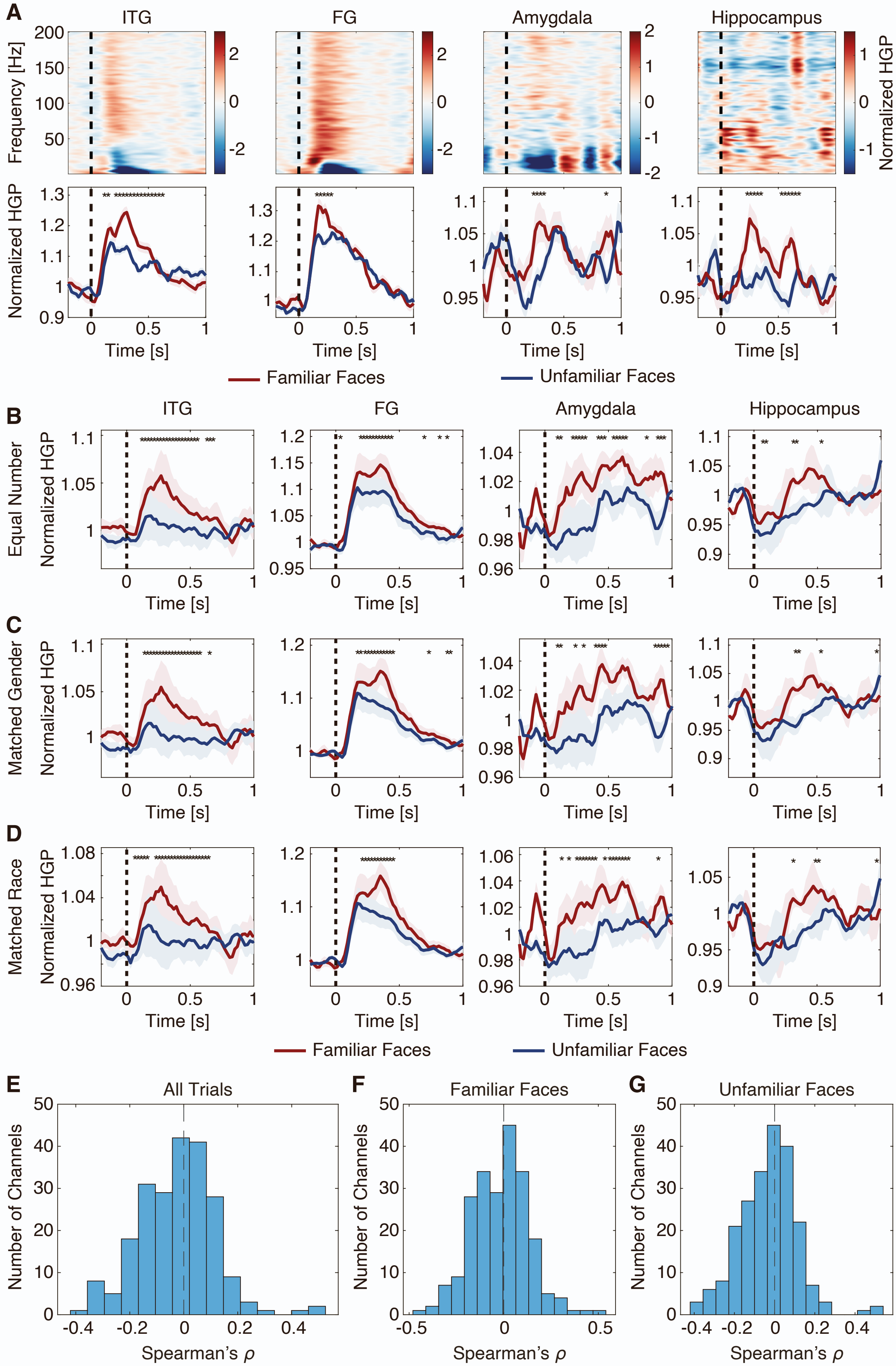

A
ITG
FG
Amygdala
Hippocampus
Frequency [Hz]
Normalized HGP
Time [s]
Familiar Faces
Unfamiliar Faces
B
Equal Number
C
Matched Gender
D
Matched Race
E
All Trials
F
Familiar Faces
G
Unfamiliar Faces
Number of Channels
Spearman's ρ

Figure S4. Differences in responses to familiar versus unfamiliar faces, Related to Figure 3. (A, B) Control analysis using an equal number of familiar and unfamiliar faces within each participant. **(C)** Control analysis using matched gender composition between familiar and unfamiliar faces. **(D)** Control analysis using matched race composition between familiar and unfamiliar faces. **(A)** Representative channels. Legend conventions as in **Figure 3B**. **(B–D)** Group results for familiarity-selective channels. Error shades denote ±SEM across channels. Asterisks indicate a significant difference between familiar and unfamiliar faces in that bin ($p < 0.05$, two-tailed paired t-test, corrected by FDR for $q < 0.05$). **(E–G)** Trial-by-trial correlations between theta-band power and high-gamma power across familiarity-coding channels. **(E)** All trials. **(F)** Familiar faces. **(G)** Unfamiliar faces.

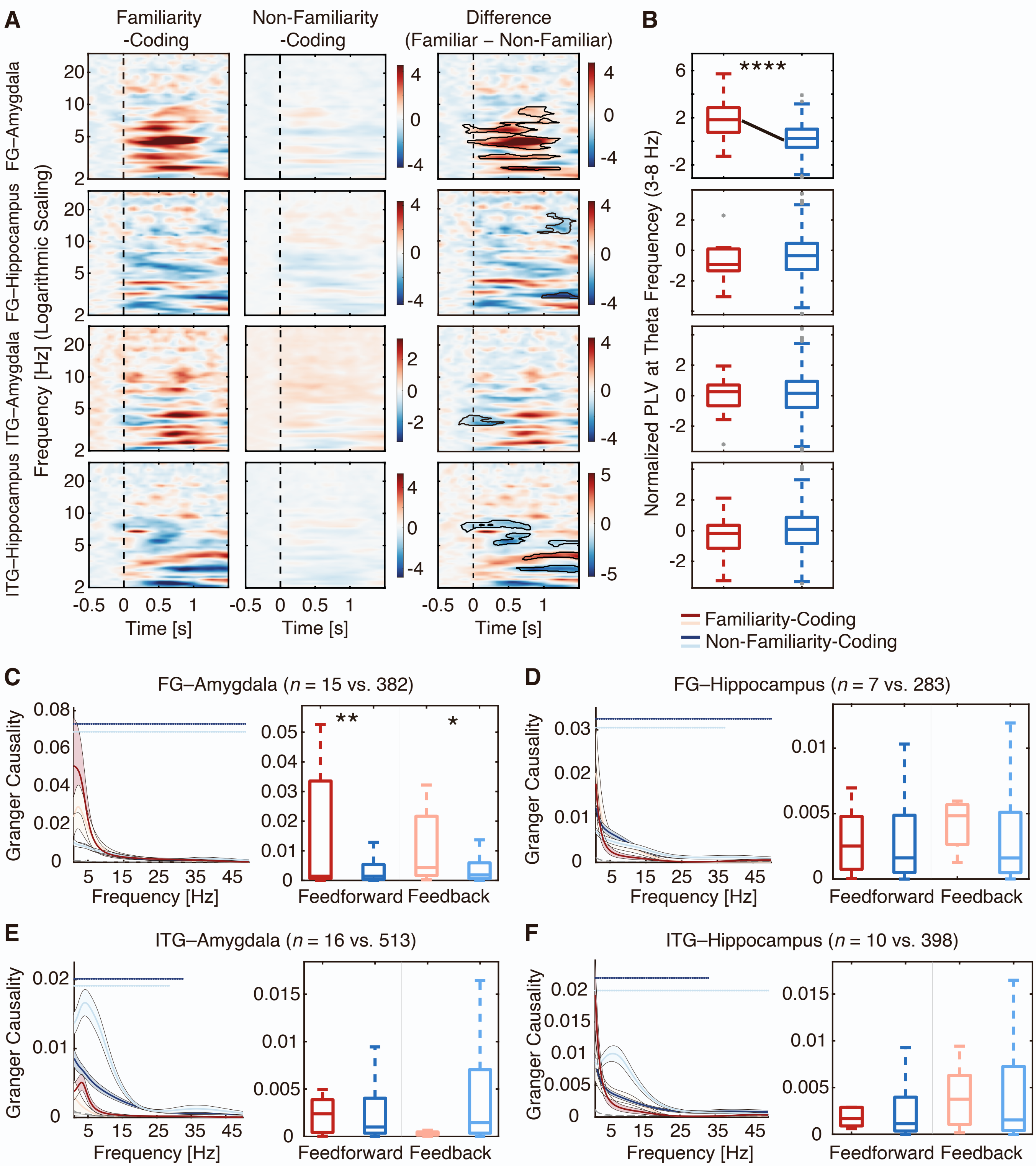

Figure S5. Functional connectivity between the VTC and MTL channels encoding familiarity, Related to Figure 4. Only channels with fewer than 5% of trials rejected were included. **(A)** Time-resolved PLV showing greater synchronization between VTC channels and MTL familiarity-coding channels compared to MTL non-familiarity-coding channels. Each row displays a combination of one

MTL subregion (amygdala or hippocampus) and one VTC subregion (FG or ITG). The PLV is normalized to the pre-trial baseline. Contours highlight areas with significant differences (see **STAR Methods**). **(B)** Average PLV within a 0–0.8-second window in the theta frequency range (3–8 Hz). Box plots show the median (central mark), the 25th and 75th percentiles (edges), and whiskers extending to non-outlier extreme values. Outliers are plotted individually. Asterisks indicate significant differences between familiarity-coding and non-familiarity-coding channel pairs, determined by bootstrapping (see **STAR Methods**). ****: $p < 0.0001$. **(C–F)** Bidirectional Granger causality (GC) between all combinations of subregions across the VTC and MTL. (left) GC across frequencies. Shaded areas represent ±SEM across channel pairs. The dashed lines indicate the 95th percentile of the null distribution, estimated via permutation. Red: GC between VTC channels and MTL familiarity-coding channels. Blue: GC between VTC channels and MTL non-familiarity-coding channels. Dark color: feedforward (from the VTC to the MTL). Light color: feedback (from the MTL to the VTC). Dots above indicate significant GC at that frequency ($p < 0.05$ across five consecutive frequencies after FDR correction). (right) Average GC within the theta frequency range (3–8 Hz), separated by feedforward and feedback GC directions. Asterisks indicate a significant difference using a two-tailed two-sample *t*-test. *: $p < 0.05$, and **: $p < 0.01$.

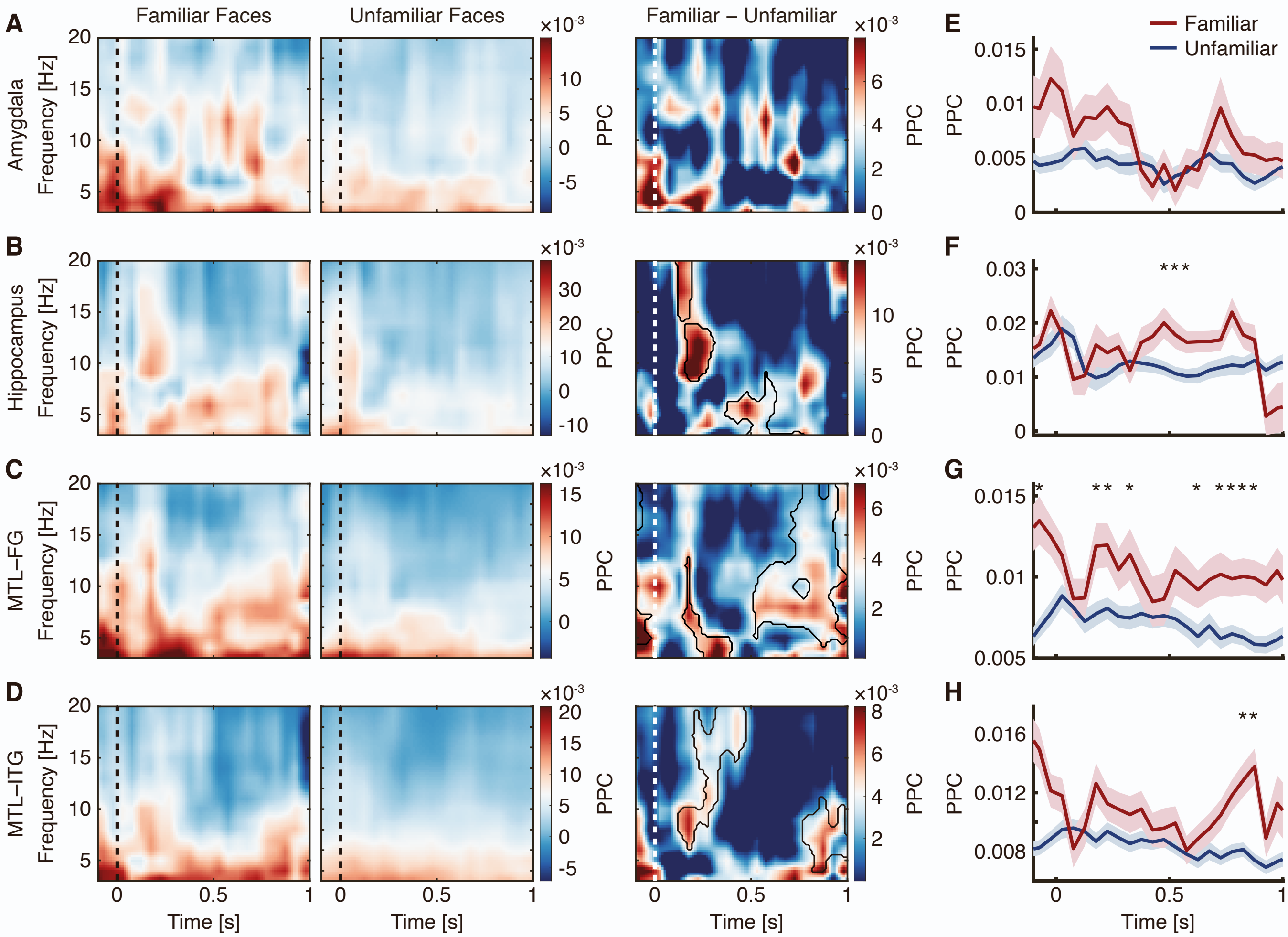

Figure S6. Time-resolved pairwise phase consistency (PPC) for spike–iEEG pairs across all subregion combinations, Related to Figure 6. (A, E) Amygdala spike–amygdala iEEG. **(B, F)** Hippocampus spike–hippocampus iEEG. **(C, G)** MTL spike–FG iEEG. **(D, H)** MTL spike–ITG iEEG. **(A–D)** Time frequency plots. **(E–H)** Mean PPC within the theta frequency range across time. Legend conventions as in **Figure 6**.

ID	Sex	Age	Race	Epilepsy diagnosis	Number of Neurons		Number of Channels			
					MTL		VTC		MTL	
					AMY	HIPP	FG	ITG	AMY	HIPP
WVU6	F	33	Caucasian	Left posterior neocortical extratemporal / parietal	10	42	n/a	n/a	n/a	n/a
					10	33	n/a	n/a	n/a	n/a
WVU7	F	28	Caucasian	Right mesial temporal	13	36	n/a	n/a	n/a	n/a
					7	21	n/a	n/a	n/a	n/a
					7	20	n/a	n/a	n/a	n/a
					2	34	n/a	n/a	n/a	n/a
WVU10	M	47	Caucasian	Right mesial temporal and neocortical temporal	26	7	n/a	n/a	n/a	n/a
					16	2	n/a	n/a	n/a	n/a
					36	29	n/a	n/a	n/a	n/a
WVU11	F	33	Caucasian	Right mesial temporal and extratemporal	25	0	n/a	n/a	n/a	n/a
					19	0	n/a	n/a	n/a	n/a
					11	25	n/a	n/a	n/a	n/a
					7	14	n/a	n/a	n/a	n/a
					0	4	n/a	n/a	n/a	n/a
WVU14	M	26	Caucasian	Bilateral amygdala/ hippocampal	27	8	n/a	n/a	n/a	n/a
					31	8	n/a	n/a	n/a	n/a
					29	45	n/a	n/a	n/a	n/a
					16	9	n/a	n/a	n/a	n/a
WVU16	F	29	Caucasian	Right temporal neocortex	24	38	n/a	n/a	n/a	n/a
					37	44	n/a	n/a	n/a	n/a
					45	47	n/a	n/a	n/a	n/a
					53	67	n/a	n/a	n/a	n/a
					42	51	n/a	n/a	n/a	n/a
WVU18	F	53	Caucasian	Left temporal	13	77	n/a	n/a	n/a	n/a
					77	24	n/a	n/a	n/a	n/a
					50	14	n/a	n/a	n/a	n/a
					37	11	n/a	n/a	n/a	n/a
WVU19	M	49	Caucasian	Right mesial temporal onset	1	33	n/a	n/a	n/a	n/a
WVU20	F	31	Caucasian	Left temporal	59	83	n/a	n/a	n/a	n/a
					49	71	n/a	n/a	n/a	n/a
					42	33	n/a	n/a	n/a	n/a

BJH025	F	45	Caucasian	Right mesial temporal	7	0	12	18	14	7
BJH026	M	40	Caucasian	Left mesial temporal	15	21	13	13	8	1
BJH032	M	27	Caucasian/ American Indian	Bilateral amygdala and hippocampus	0	7	20	17	9	13
					0	2	20	17	9	12
BJH051	F	41	Caucasian	Left amygdala and hippocampus	13	13	9	11	6	2
BJH054	F	36	Caucasian	Left temporal and fusiform	12	12	11	27	7	7
BJH065	M	39	American Indian/ Alaskan Native	Right temporal	n/a	n/a	8	2	7	3
					n/a	n/a	9	10	7	1
BJH069	M	26	Caucasian	Left amygdala, hippocampus, medial temporal pole and entorhinal cortex	n/a	0	7	10	5	10
					n/a	0	7	10	5	10
					n/a	15	7	10	5	10
					n/a	22	7	11	5	10
BJH072	F	27	Caucasian	Left amygdala, hippocampus and entorhinal cortex	n/a	n/a	11	14	3	8
BJH074	M	30	Caucasian	Right amygdala	n/a	22	5	19	11	6
BJH075	M	25	Black/ African American	Left hippocampus	n/a	n/a	8	8	9	0
					n/a	n/a	8	8	9	0
BJH077	M	47	Caucasian	Bilateral amygdala and hippocampus	n/a	n/a	5	6	9	6
BJH078	F	57	Black/ African American	Left hippocampus and temporal	0	0	9	13	4	4
BJH080	F	42	Caucasian	Left temporal, amygdala and hippocampus	0	0	1	3	1	1
					0	0	5	4	4	2
	Sum				868	1044	182	231	137	113

Table S1. Patients and sessions for single-neuron and iEEG recordings, Related to Figure 1 and Figure 3. Each row represents a session. VTC: ventral temporal cortex. MTL: medial temporal lobe. FG: fusiform gyrus. ITG: inferior temporal gyrus. AMY: amygdala. HIPP: hippocampus. n/a: not available. “n/a” indicates that no electrodes were implanted in a given brain structure. “0” indicates that electrodes were implanted in a brain structure, but no viable neurons were identified (for the neuron column), or that all iEEG channels in that brain area were rejected (for the channel column).
